# A Straightforward Diphenylmethyl Protection Method and Deprotection of Some Pyrimidine Nucleosides

**DOI:** 10.3390/molecules18078524

**Published:** 2013-07-18

**Authors:** Milind Saudi, Arthur van Aerschot

**Affiliations:** Medicinal Chemistry, Rega Institute for Medical Research, KU Leuven, Minderbroedersstraat 10, Leuven 3000, Belgium; E-Mail: milind.saudi@rega.kuleuven.be

**Keywords:** hydroxyl protecting group, palladium catalyst, nucleoside, diphenyl methyl

## Abstract

Benzhydryl protection of primary and secondary alcohols has been reported previously via reaction with metal alcoholates. Our aim was to find generally useful and very mild conditions for the alcoholic protection and deprotection of nucleosides with the diphenylmethyl group. This was accomplished for some pyrimidine nucleosides using PdCl_2_ as the transition metal catalyst, and with optimization yields of 70–90% have been achieved. A lack of solubility of other nucleosides hampers its more general use.

## 1. Introduction

Nucleoside and nucleotide chemistry generally involves multistep synthesis protocols, where selective and efficient protection and deprotection reactions have always played a central role. The criteria involved in the choice of protecting groups are based on high yielding protection and deprotection steps, chemoselectivity and orthogonality with other protecting and functional groups. For example, various acyl, benzyl, trityl, triethylsilyl [1], and *tert*-butyldimethylsilyl ethers [2] have found common use for selective protection of primary/secondary alcohols, particularly in carbohydrate and nucleoside/nucleotide chemistry. To our knowledge, only very few specific reviews for nucleoside protection are available [3,4]. For a complete overview of alcohol protecting groups readers may consult Greene and Wuts [5].

Due to their orthogonality regarding deprotection conditions, the 5′-, 3′- and 2′-hydroxyl groups of nucleosides are usually temporarily acylated or converted to either silyl or alkyl ethers for protection, of which the benzyl and trityl groups represent the most popular ethers [6]. The trityl moiety can be readily introduced even on a secondary alcohol under neutral conditions in presence of a hydrogen chloride scavenger like pyridine or triethylamine. In contrast, *O*-alkylation with a benzyl or diphenyl methyl group generally requires increased activity of the alcohol via deprotonation [7], or activation of the benzyl moiety as a trichloroacetimidate combined with acidic reaction conditions [8]. In some instances, robust protection is mandatory as with a benzyl moiety. The latter however requires hydrogenation conditions for its removal. An alternative protecting strategy therefore is warranted. Hence, we now want to report on metal-catalyzed conditions for protection and deprotection of nucleoside analogues using the diphenylmethyl (DPM) ether group.

Synthesis of DPM ethers using many different strategies has been previously reported, but this protecting group is not very common. As for most benzyl-type ethers, its introduction usually involves nucleophilic substitution of chloro- or bromodiphenylmethane using sodium hydride-generated alkoxides [9]. However, diphenylmethanol in the presence of either a Brønsted acid, Lewis acid, or supported acids likewise has been shown to form DPM ethers. Mechanistically the reaction occurs either via intimate ion-molecule pairs, via solvent separated ion-molecule pairs or via benzhydryl carbocations [10]. A few other methods can nevertheless be found in the literature. These include reagents such as diphenyl-methylphosphate–trifluoroacetic acid [11], diphenylmethyldiazomethane [12,13], or the use of orthoformates [14] at high temperature.

Many other strategies involve metal-catalysed reactions. Ishii, in 2003, reported the first use of Yb(OTf)_3_, Sc(OTf)_3_ and Hf(OTf)_3_ as Lewis acids for the direct reaction of alcohols. Quite interestingly, Ishii found that phenylethanol is in equilibrium with its diether during the reaction with La(OTf)_3_ as catalyst [15]. Pale and co-workers reported the use of palladium (II) chloride as a catalyst [16,17]. Later they reported CuBr_2_ to be an even better catalyst [18]. Recently, an effective method for the allylation of heteroarenes has also been catalysed by PdCl_2_ [19]. Baba and his co-workers reported the use of InCl_3_ in the direct substitution of allylic alcohols, benzylic alcohols and benzhydrylic alcohols with nucleophiles. Mechanistic investigation showed that the ether was obtained by the action of In (III) salts [20]. Highly regioselective catalytic amination of Baylis-Hillman adduct with aromatic amines has been promoted by the Lewis acid In(OTf)_3_ [21].

As for any benzyl-type ether, deprotection of DPM ethers is mostly achieved under hydrogenation conditions [22], but many other protocols have been reported, including acidolysis [23] and electrolytic reduction [24]. Our research focused on finding a simpler and environmentally acceptable procedure for protection and deprotection of DPM ethers specifically for nucleoside chemistry. We therefore adopted a strategy using transition metals as the catalyst with preference for Pd salts.

## 2. Results and Discussion

**Scheme 1:**
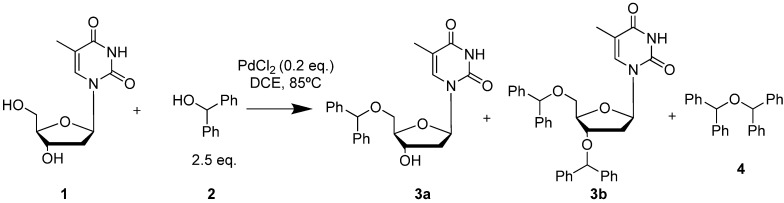
Protection of both hydroxyl groups of thymidine as diphenylmethyl ether.

**Scheme 2:**
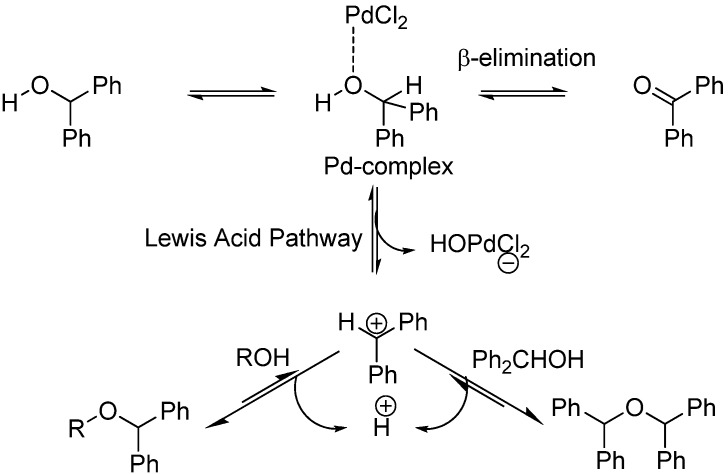
Mechanism for diphenylmethyl ether protection.

In search for the best catalyst, we allowed thymidine (**1**) at a scale of 100 mg to react with diphenylmethanol (DPM-OH) in dichloroethane (DCE) in presence of 0.2 equivalents (eq) of various metal salts. Initially, as the rate of reaction was too low at room temperature, we decided to heat the reaction to 85 °C. As shown in Scheme 1, we expected three different products, the mono- and di-*O*-protected thymidines **3a** and **3b**, and ether **4**, resulting from dimerization of diphenylmethanol. However, using 2.5 eq of DPM-OH and following heating overnight, thymidine **1** was cleanly converted to its protected form **3b** in 87% yield with only trace formation of the unwanted ether **4** (Table 1). Likewise formation of benzophenone was not observed. This result indicated that there is complex formation by coordination between the Pd catalyst and diphenylmethanol in which the Lewis acid character of the Pd-complex outbalances the β-elimination pathway (Scheme 2).

**Table 1:** Comparison of catalyst effectiveness for formation of bis-benzhydryl thymidine (**3b**).

Exp.	Catalyst	Recovered 1 (%)	Recovered 2 (%)	Yield 3b (%) *^a^*	Yield 4 (%)
1	PdCl_2_	-	6	87	Trace
2	NiCl_2_	95	94	2	-
3	CuCl_2_·2H_2_O	43	0	55 (24 h)	Trace
4	Pd(OAc)_2_	10	0	65	-
5	Ni(OAc)_2_	90	0	0	Trace
6	Cu(OAc)_2_	32	-	45	Trace

*^a^* Reactions on 100 mg scale were run in refluxing DCE for 16 h with 0.2 eq of the metal catalyst and 2.5 eq of DPM-OH.

As we wanted to favor the Lewis acid pathway, we further evaluated catalysts which can form more acidic salt complexes, e.g., nickel being located in the same column as palladium in the Mendeleev periodic table. Unfortunately, when NiCl_2_ was used we did not obtain the expected product, and even after 48 h 95% of the starting material was recovered. Reaction in presence of CuCl_2_·2H_2_O offered the desired product, albeit in low yield. This metal salt screening showed that PdCl_2_ was the most effective catalyst for protection of both hydroxyl groups in **1** as their diphenylmethyl ethers, affording almost quantitative yields of the desired product (Table 1).

We also evaluated the effectiveness of metal-catalysed DPM protection in different solvents with thymidine as substrate. As expected, non-polar solvents like dichloroethane, being non-coordinating, proved to be the best and reactions looked very clean on TLC. Formation of diphenylmethanol dimer remained below 5%. On the other hand, polar solvents like DMF led to messy reactions in which the desired product could not be isolated (Table 2). Finally, when carried out on a 2 mmol scale in DCE, 70% of the desired **3b** was isolated.

**Table 2:** Solvent influence on DPM ether formation at either 85 °C or boiling temperature.

Entry	Solvent	Time (h)	Yield 3b (%)
1	DCE	16	87
2	Acetonitrile	48	47
3	THF	48	42
4	Dioxane	16	Degradation
5	DMF	16	Degradation

Having optimized the conditions for DPM protection for **1**, we decided to evaluate this reaction for other nucleosides under similar conditions. Using 2.5 eq of DPM-OH, both 2′-deoxyuridine and 5-fluoro-2′-deoxyuridine reacted cleanly to afford the ethers **6b** and **7b** in 88% and 85% yield, respectively. Moreover selective protection of the 5′ primary alcohol proved feasible for 2′-deoxynucleosides using only 1.2 eq of DPM-OH with heating at 85 °C, and afforded around 65% for each of the mono-*O*-alkylated analogues **3a**, **6a** and **7a** (Table 3) with likewise 67% yield for **3a** on a 2 mmol scale. Only trace amounts of the bis-diphenylmethyl ether analogues were isolated. Unfortunately, reaction of the ribonucleosides uridine and 5-fluorouridine with 1.2 eq of DPM-OH only afforded a trace of the desired 5′-*O*-protected nucleoside (Table 3, experiments 7 and 10, respectively). Using 2.5 eq of DPM-OH on both uridine analogues, more than 80% of the bisalkylated derivatives were obtained as a mixture of the 2′,5′- and 3′,5′-*O*-alkylated conformers (Table 3, experiments 8 and 11, respectively). Finally, per-*O*-alkylation of ribonucleosides in about 80% yield proved feasible by increasing the incoming DPM-OH ratio to 3.7 eq. This proves much more difficult with the sterically more demanding trityl moieties [25]. Separation of both bis-diphenylmethyl ethers (2′,5′- and 3′,5′-derivative) was cumbersome, but was achieved by using appropriate solvent polarity during column chromatography. The correct position of the diphenylmethyl moiety was determined using 2D-NMR techniques (COSY, HSQC), with the 2′,5′-analogue proving slightly more lipophilic than the 3′,5′-analogue.

As reported before [17], the reaction is believed to occur via formation of the latter benzhydryl carbocation. As shown in Scheme 2, coordination of the hydroxyl oxygen atom to the mild Lewis acid Pd^II^ is highly likely. Following coordination, there may be transfer of the hydroxyl group to the palladium affording the benzhydryl carbocation.

**Scheme 3:**
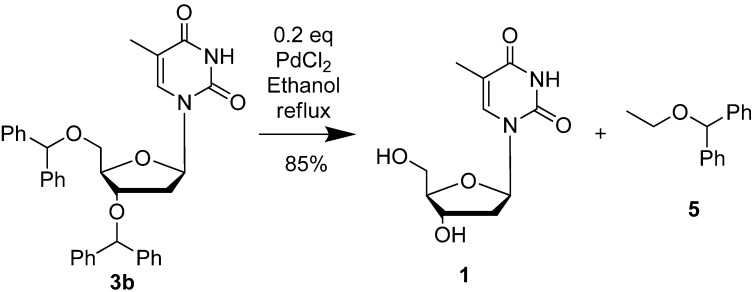
Deprotection of the DPM ether moiety in refluxing ethanol.

As shown in Scheme 3, deprotection of synthesized 3′,5′-di-*O*-benzhydryl-thymidine **3b** was successfully achieved by only changing solvent. Following heating of **3b** in ethanol at 85 °C with catalytic PdCl_2_ (0.2 eq) for 16 h, it was cleanly converted to thymidine.

**Table 3:** Synthesis of different benzhydryl protected nucleosides.

Entry	Substrate	Eq DPM-OH	Product	Yield (%)	
1	thymidine	1.2	5′-*O*-benzhydryl-thymidine **3a**	67	
2		2.5	3′,5′-di-*O*-benzhydryl-thymidine **3b**	87	
3	2′-deoxyuridine	1.2	5′-*O*-benzhydryl-2′-deoxyuridine **6a**	65	
4		2.5	3′,5′-di-*O*-benzhydryl-2′-deoxyuridine **6b**	88	
5	5-fluoro-2′-deoxyuridine	1.2	5′-*O*-benzhydryl-5-fluoro-2′-deoxyuridine **7a**	64	
6		2.5	3′,5′-di-*O*-benzhydryl-5-fluoro-2′-deoxyuridine **7b**	85	
7	uridine	1.2	5′-*O*-benzhydryl-uridine **8a**	Trace	
8		2.5	2′,5′-di-*O*-benzhydryl-uridine **8b**	54	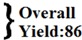
			3′,5′-di-*O*-benzhydryl-uridine **8c**	32	
9		3.7	2′,3′,5′-tris-*O*-benzhydryl-uridine **8d**	81	
10	5-fluorouridine	1.2	5′-*O*-benzhydryl-5-fluorouridine **9a**	Trace	
11		2.5	2′,5′-di-*O*-benzhydryl-5-fluorouridine **9b**	59	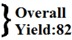
			3′,5′-di-*O*-benzhydryl-5-fluorouridine **9c**	23	
12		3.7	2′,3′,5′-tris-*O*-benzhydryl-5-fluorouridine **9d**	79	

The resulting byproduct ethoxydiphenylmethane **5** was also isolated and characterized by ^1^H-NMR showing a singlet at 5.44δ, a quartet at 3.61δ and a triplet at 1.35δ. Deprotection was also attempted using CuBr_2_ in catalytic amounts, but even after refluxing for 16 h, presence of the starting material was still observed along with some degraded material (Table 4). The use of CuBr_2_ however has been reported before for clean cleavage of bis-(methoxyphenyl)methyl ether moieties [18]. The latter are much more prone to cleavage in agreement with the strongly reduced acid stability of dimethoxytrityl ether in comparison with trityl moieties.

**Table 4:** Deprotection of DPM ethers in ethanol at 85 °C.

Entry	Catalyst	Time (h)	Yield of 1 (%) ^a^
1	PdCl_2_	16	85
2	CuBr_2_	16	43

^a^ Reaction on 100 mg scale in refluxing ethanol for 16 h with 0.2 eq of metal catalyst.

As reported before [16], introduction of DPM ether is compatible with the presence of different protecting groups like acyl groups, silyl and benzyl ethers or ester moieties. In our hands, following overnight treatment of **3b** with conc. ammonia:MeOH (1:1) or 2M NaOH:dioxane (1:1) no reaction was observed. The compounds likewise proved relatively stable in acidic conditions showing no reaction at RT in 80% acetic acid with overnight treatment, but starting to degrade after heating for 4 h at 80 °C. Finally, a 3% trichloroacetic acid solution in DCE afforded deprotection only after 15 h of reaction, with 50% benzhydryl cleavage after 6 h. Unfortunately, use of the DPM group seems limited to a small series of pyrimidine nucleosides. When evaluating cytidine, adenosine or guanosine nucleosides, the lack of solubility of the starting compounds prevented reaction in DCE while in more polar solvents, degradation was noticed with time. In an attempt to remediate the problem, prior persilylation with bis(trimethylsilyl)acetamide afforded soluble substrates for 2′-deoxycytidine and guanosine, but upon addition of DPM-OH and PdCl_2_, rapid precipitation occurred and no alkylated nucleosides could be obtained. Finally, adding small amounts of polar solvents like NMP to improve the solubility in DCE prevented reaction as of likely complexation of the PdCl_2_. In conclusion, straightforward introduction and ease of removal of the DPM ether on uridine analogues, promised to make a nice addition to the nucleosidic protecting group repertoire, but unfortunately, the lack of solubility of other nucleosides hampers its general use.

## 3. Experimental

### 3.1. General

Reagents and solvents were purchased from commercial suppliers (Acros, Sigma-Aldrich, Bachem, Novabiochem) and used as provided, unless indicated otherwise. All the solvents were of analytical grade and were stored over 4Å molecular sieves. Reactions were carried out in oven-dried glassware under a nitrogen atmosphere with stirring at 85 °C.

^1^H and ^13^C-NMR spectra of the compounds dissolved in CDCl_3_, MeOD or DMSO-*d_6_* were recorded on a Bruker UltraShield Avance 300 MHz or 600 MHz spectrometer. The chemical shifts are expressed as δ values in parts per million (ppm), using the residual solvent peaks (CDCl_3_: ^1^H, 7.26 ppm; ^13^C, 77.16 ppm; MeOD: ^1^H, 3.31 ppm; ^13^C, 49.00 ppm) as a reference. Coupling constants are given in Hertz (Hz). The peak patterns are indicated by the following abbreviations: bs = broad singlet, d = doublet, m = multiplet, q = quadruplet, s = singlet and t = triplet. High resolution mass spectra were recorded on a quadrupole time-of-flight mass spectrometer (Q-Tof-2, Micromass, Manchester, UK) equipped with a standard ESI interface; samples were infused in 2-propanol/H2O (1:1) at 3 µL min^−1^.

For TLC, precoated aluminium sheets were used (Merck, Silica gel 60 F254). The spots were visualized by UV light at 254 nm. Column chromatography was performed on ICN silica gel 60A° 40–60 µM.

### 3.2. General Procedure for Protection

To a solution of the respective nucleoside (100 mg) and diphenylmethanol in dichloroethane (5 mL/mmol) was added palladium chloride (0.2 eq). The reaction mixture was heated at 85 °C under an argon atmosphere for 16 h or until disappearance of the starting materials as monitored by TLC. The solvent was removed *in vacuo* and the crude mixture obtained was purified by column chromatography to afford the desired compound.

*5′-O-Benzhydryl-thymidine* (**3a**). White solid; TLC (DCM/MeOH 9:1): R*_f_* = 0.70. ^1^H-NMR (300 MHz, CDCl_3_): δ 8.96 (bs, 1H, NH), 7.55 (s, 1H), 7.54–7.28 (m, 10H), 6.50–6.45 (m, 1H, H-1′), 5.44 (s, 1H, CH), 4.63 (s, 1H), 4.14–4.13 (m, 1H), 3.80–3.76 (m, 1H, H-5′/H-5′′), 3.68–3.63 (m, 1H, H-5′/H-5′′), 2.79 (bs, 1H, OH), 2.46–2.39 (m, 1H, H-2′/H-2′′), 2.36–2.27 (m, 1H, H-2′/H-2′′), 1.41 (s, 3H). ^13^C-NMR (75 MHz, CDCl_3_) δ: 163.4, 150.2, 141.2, 141.0, 135.4, 128.4, 127.7, 127.5, 126.6, 126.0, 111.0, 85.8, 84.6, 84.5, 72.5, 68.8, 40.7, 11.4. HRMS calcd for C_23_H_24_N_2_O_5_ [M+Na]^+^: 431.1577; found:431.1576.

*3′,5′-di-O-Benzhydryl-thymidine* (**3b**). White solid; TLC (ethyl acetate/hexane 1:1): R*_f_* = 0.70. ^1^H-NMR (300 MHz, CDCl_3_): δ 9.28 (bs, 1H, NH), 7.50 (s, 1H), 7.49–7.19 (m, 20H), 6.53–6.48 (m, 1H, H-1′), 5.47 (s, 1H, CH), 5.37 (s, 1H, CH), 4.43–4.39 (m, 1H), 4.33–4.30 (m, 1H), 3.77–3.72 (m, 1H, H-5′/H-5′′), 3.53–3.49 (m, 1H, H-5′/H-5′′), 2.62–2.54 (m, 1H, H-2′/H-2′′), 2.24–2.15 (m, 1H, H-2′/H-2′′), 1.42 (s, 3H). ^13^C-NMR (75 MHz, CDCl_3_): δ 163.6, 150.2, 141.3, 141.2, 141.1, 141.0, 135.2, 128.4, 128.3, 128.3, 128.2, 127.6, 127.5, 127.5, 126.8, 126.7, 126.5, 126.1, 110.9, 84.8, 84.3, 83.8, 82.1, 77.2, 77.2 68.7, 38.3, 11.4. HRMS calcd for C_36_H_34_N_2_O_5_ [M+H]^+^: 575.2540; found: 575.2540.

*5′-O-Benzhydryl-2′-deoxyuridine* (6a). White solid; TLC (ethyl acetate/hexane 1:1): R*_f_* = 0.30. ^1^H-NMR (600 MHz, CDCl_3_): δ 8.35 (bs, 1H, NH), 7.77 (d, 1H, *J =* 7.8 Hz), 7.36–7.26 (m, 10H), 6.39 (t, 1H, H-1′, *J =* 6.6 Hz), 5.39 (s, 1H, CH), 5.21–5.19 (m, 1H), 4.64–4.62 (m, 1H), 4.10 (s, 1H), 3.81–3.78 (m, 1H, H-5′/H-5′′), 3.72–3.70 (m, 1H, H-5′/H-5′′), 2.41–2.38 (m, 1H, H-2′/H-2′′), 2.29–2.24 (m, 1H, H-2′/H-2′′), 2.05 (d, 1H, OH, *J =* 3.6 Hz). ^13^C-NMR (150 MHz, CDCl_3_): δ 162.7, 150.0, 141.2, 140.9, 140.3, 128.7, 128.7, 128.1, 128.0, 126.8, 126.7, 102.1, 86.0, 85.1, 85.0, 72.3, 68.9, 59.5, 41.3. HRMS calcd for C_22_H_22_N_2_O_5_ [M+Na]^+^: 417.1421; found: 417.1421.

*3′,5′-di-O-Benzhydryl-2′-deoxyuridine (**6b**)*. White solid; TLC (ethyl acetate/hexane 1:1): R*_f_* = 0.70. ^1^H-NMR (600 MHz, CDCl_3_): δ 8.32 (bs, 1H, NH), 7.70 (d, 1H, *J =*8.4 Hz), 7.36–7.18 (m, 20H), 6.41 (t, 1H, H-1′, *J =*6.3 Hz), 5.44 (s, 1H, CH), 5.31 (s, 1H, CH), 5.19–5.15 (m, 1H), 4.39–4.36 (m, 1H), 4.31–4.28 (m,1H), 3.77–3.73 (m, 1H, H-5′/H-5′′), 3.57–3.53 (m, 1H, H-5′/H-5′′), 2.56–2.50 (m, 1H, H-2′/H-2′′), 2.16–2.07 (m, 1H, H-2′/H-2′′). ^13^C-NMR (150 MHz, CDCl_3_): δ 162.8, 150.0, 141.4, 141.3, 141.2, 140.8, 140.2, 128.7, 128.6, 128.5, 128.0, 127.9, 127.8, 127.1, 127.0, 126.7, 126.6, 102.1, 85.3, 84.9, 84.3, 82.4, 69.0, 38.8. HRMS calcd for C_35_H_32_N_2_O_5_ [M+H]^+^: 561.2383; found: 561.2377.

*5′-O-Benzhydryl-5-fluoro-2′-deoxyuridine* (**7a**). White solid; TLC (ethyl acetate/hexane 1:1): R*_f_* = 0.20. ^1^H-NMR (300 MHz, CDCl_3_): δ 8.39 (bs, 1H, NH), 7.95 (d, 1H, *J =* 6.3 Hz), 7.37–7.28 (m, 10H), 6.40–6.35 (m, 1H, H-1′), 5.43(s, 1H, CH), 4.64–4.62 (m, 1H), 4.15–4.13 (m, 1H), 3.87–3.82 (m, 1H, H-5′/H-5′′), 3.70–3.66 (m, 1H, H-5′/H-5′′), 2.48–2.40 (m, 1H, H-2′/H-2′′), 2.31–2.22 (m, 1H, H-2′/H-2′′), 1.95 (bs, 1H, OH). ^13^C-NMR (75 MHz, CDCl_3_): δ 156.6, 156.4, 148.5, 141.2, 141.0, 140.8, 139.6, 128.8, 128.1, 128.0, 126.8, 126.6, 124.3, 124.1, 86.3, 85.6, 85.3, 72.6, 69.0, 41.3. HRMS calcd for C_22_H_21_FN_2_O_5_ [M+Na]^+^: 435.1327; found: 435.1328.

*3′,5′-di-O-Benzhydryl-5-fluoro-2′-deoxyuridine* (**7b**). White solid; TLC (ethyl acetate/hexane 1:1): R*_f_* = 0.70. ^1^H-NMR (600 MHz, CDCl_3_): δ 8.42 (bs, 1H, NH), 7.86 (d, 1H, *J=* 6Hz), 7.34–7.15 (m, 20H), 6.37–6.34 (m, 1H, H-1′), 5.40(s, 1H, CH), 5.29 (s, 1H, CH), 4.33–4.28 (m, 2H), 3.75–3.73 (m, 1H, H-5′/H-5′′), 3.48–3.46 (m, 1H, H-5′/H-5′′), 2.56–2.52 (m, 1H, H-2′/H-2′′), 2.09–2.04 (m, 1H, H-2′/H-2′′). ^13^C-NMR (150 MHz, CDCl_3_): δ 156.6, 156.4, 141.4, 141.3, 141.2, 140.9, 139.6, 128.7, 128.6, 128.5, 128.5, 128.0, 127.9, 127.8, 127.1, 127.0, 124.3, 124.1, 85.8, 85.1, 84.5, 82.5, 76.8, 69.0, 38.7. HRMS calcd for C_35_H_31_FN_2_O_5_ [M+Na]^+^: 601.2109; found: 601.2098.

*2′,5′-di-O-Benzhydryl-uridine* (**8b**). White solid; TLC (ethyl acetate/hexane 1:1): R*_f_* = 0.60. ^1^H-NMR (600 MHz, CDCl_3_): δ 8.78 (bs, 1H, NH), 7.65 (d, 1H, *J =* 8.2 Hz), 7.37–7.19 (m, 20H), 6.22 (d, 1H, H-1′, *J =* 4.2 Hz), 5.80 (s, 1H, CH), 5.37 (s, 1H, CH), 4.93–4.91 (m, 1H), 4.37–4.34 (m, 1H), 4.23–4.21 (m, 1H), 4.15 (t, 1H, *J =* 4.8 Hz), 3.85–3.83 (m, 1H, H-5′/H-5′′), 3.71–3.69 (m, 1H, H-5′/H-5′′), 2.75 (d, 1H, OH, *J =* 6 Hz). ^13^C-NMR (150 MHz, CDCl_3_): δ 162.7, 149.9, 140.9, 140.6, 140.4, 139.9, 139.6, 128.5, 128.4, 128.3, 127.9, 127.7, 126.9, 126.6, 126.6, 126.4, 101.7, 86.6, 84.8, 83.7, 82.6, 79.5, 69.2, 67.7. HRMS calcd for C_35_H_32_N_2_O_6_ [M+H]^+^: 577.2333; found : 577.2336.

*3′,5′-ditri-O-Benzhydryl-uridine* (**8c**). White solid; TLC (ethyl acetate/hexane 1:1): R*_f_* = 0.40. ^1^H-NMR (600 MHz, CDCl_3_): δ 8.39 (bs, 1H, NH), 7.63 (d, 1H, *J =* 8.2 Hz), 7.39–7.17 (m, 20H), 6.02 (d, 1H, H-1′, *J =* 4.2 Hz), 5.52 (s, 1H, CH), 5.26 (s, 1H, CH), 5.13–5.11 (m, 1H), 4.27–4.25 (m, 2H), 4.20–4.17 (m, 1H), 3.72–3.70 (m, 1H, H-5′/H-5′′), 3.41–3.39 (m, 1H, H-5′/H-5′′), 2.95 (d, 1H, OH, *J =* 7.2 Hz). ^13^C-NMR (150 MHz, CDCl_3_): δ 162.4, 150.0, 140.7, 140.4, 140.2, 139.7, 128.5, 128.4, 128.3, 127.9, 127.9, 127.8, 127.6, 126.8, 126.6, 126.4, 126.3, 101.9, 89.0, 84.6, 83.6, 81.5, 75.9, 74.0, 67.8. HRMS calcd for C_35_H_32_N_2_O_6_ [M+H]^+^: 577.2333; found: 577.2330.

*2′,3′,5′-tri-O-Benzhydryl-uridine* (**8d**): White solid; TLC (ethyl acetate/hexane 1:1): R*_f_* = 0.80. ^1^H-NMR (600 MHz, CDCl_3_): δ 8.63 (bs, 1H, NH), 7.63 (d, 1H, *J =* 8.4 Hz), 7.27–7.03 (m, 30H), 6.23 (d, 1H, H-1′, *J =* 3 Hz), 5.75(s, 1H, CH), 5.25 (s, 1H, CH), 5.21 (s, 1H, CH), 4.78–4.76 (m, 1H), 4.46 (d, 1H, *J =* 6 Hz), 4.14 (t, 1H, *J =* 5.4 Hz), 4.06–4.04 (m, 1H), 3.78–3.76 (m, 1H, H-5′/H-5′′), 3.56–3.54 (m, 1H, H-5′/H-5′′). ^13^C-NMR (150 MHz, CDCl_3_): δ 162.5, 149.5, 141.3, 140.7, 140.6, 140.5, 139.6, 128.5, 128.4, 128.3, 128.2, 128.1, 128.0, 127.9, 127.7, 127.6, 127.6, 127.5, 127.4, 127.3, 127.2, 127.1, 126.5, 126.4, 126.4, 126.3, 101.3, 87.4, 84.5, 81.8, 81.8, 77.7, 72.4, 67.0. HRMS calcd for C_48_H_42_N_2_O_6_ [M+H]^+^: 743.3115; found: 743.3107.

*2′,5′-di-O-Benzhydryl-5-fluorouridine* (**9b**). White solid; TLC (ethyl acetate/hexane 1:1): R*_f_* = 0.60. ^1^H-NMR (600 MHz, CDCl_3_): δ 8.61 (bs, 1H, NH), 7.68 (d, 1H, *J =* 12.6 Hz), 7.36–7.22 (m, 20H), 6.21–6.20 (m, 1H, H-1′), 5.67 (s, 1H, CH), 5.38 (s, 1H, CH), 4.33–4.31 (m, 1H), 4.24–4.22 (m, 1H), 4.17–4.15 (m, 1H), 3.86–3.83 (m, 1H, H-5′/H-5′′), 3.65–3.62 (m, 1H, H-5′/H-5′′), 2.75 (d, 1H, OH, *J =* 5.4 Hz). ^13^C-NMR (150 MHz, CDCl_3_): δ 148.6, 140.9, 140.6, 140.4, 140.3, 139.9, 139.0, 128.5, 128.4, 128.3, 127.9, 127.7, 126.8, 126.6, 126.5, 126.3, 123.5, 123.3, 86.6, 85.2, 83.9, 83.2, 79.4, 69.5, 68.1. HRMS calcd for C_35_H_31_FN_2_O_6_ [M+Na]^+^: 617.2058; found: 617.2062.

*3′,5′-ditri-O-Benzhydryl-5-fluorouridine* (**9c**): White solid; TLC (ethyl acetate/hexane 1:1): R*_f_* = 0.40. ^1^H-NMR (600 MHz, CDCl_3_): δ 8.38 (bs, 1H, NH), 7.78 (d, 1H, *J =* 7.2 Hz), 7.36–7.16 (m, 20H), 6.02–6.00 (m, 1H, H-1′), 5.50 (s, 1H, CH), 5.27 (s, 1H, CH), 4.25–4.23 (m, 2H), 4.20–4.17 (m, 1H), 3.73–3.70 (m, 1H, H-5′/H-5′′), 3.32–3.30 (m, 1H, H-5′/H-5′′), 2.94 (d, 1H, OH, *J =* 9.6 Hz). ^13^C-NMR (150 MHz, CDCl_3_): δ 156.2, 155.9, 148.5, 141.2, 140.4, 140.3, 140.1, 139.3, 128.5, 128.4, 128.3, 128.0, 127.9, 127.8, 127.6, 126.8, 126.6, 126.5, 126.4, 123.8, 123.5, 89.0, 84.8, 83.8, 81.9, 76.3, 74.1, 67.9. HRMS calcd for C_35_H_31_FN_2_O_6_ [M+Na]^+^: 617.2058; found: 617.2061.

*2′,3′,5′-tri-O-Benzhydryl-5-fluorouridine* (**9d**). White solid; TLC (ethyl acetate/hexane 1:1): R*_f_* = 0.80. ^1^H-NMR (600 MHz, CDCl_3_): δ 8.55 (d, 1H, NH, *J =* 5.4 Hz), 7.64 (d, 1H, *J =* 7.2 Hz), 7.37–7.06 (m, 30H), 6.23 (m, 1H, H-1′), 5.60 (s, 1H, CH), 5.34 (s, 1H, CH), 5.23 (s, 1H, CH), 4.43–4.41 (m, 1H), 4.16–4.13 (m, 1H), 4.08–4.05 (m, 1H), 3.79–3.76 (m, 1H, H-5′/H-5′′), 3.46–3.44 (m, 1H, H-5′/H-5′′). ^13^C-NMR (150 MHz, CDCl_3_): δ 156.7, 148.6, 141.5, 141.1, 141.0, 140.9, 140.8, 140.6, 139.1, 128.6, 128.5, 128.4, 128.3, 128.0, 127.9, 127.8, 127.7, 127.5, 127.3, 127.2, 127.0, 126.9, 126.8, 126.7, 123.8, 123.5, 87.4, 85.1, 82.7, 82.5, 82.4, 78.3, 73.2.4, 67.9. HRMS calcd for C_48_H_41_FN_2_O_6_ [M+H]^+^: 761.3021; found: 761.3037.

### 3.3. General Procedure for Deprotection

To a solution of 3′,5′-di-*O*-benzhydryl-thymidine **3b** (100 mg) in ethanol (5 mL/mmol) was added palladium chloride (0.2 eq). The reaction mixture was heated at 85 °C for 16 h until disappearance of the starting material monitoring by TLC. The solvent was removed *in vacuo* and the crude mixture obtained was purified by column chromatography affording compound **5** and recovered **1** in 85% yield.

*Ethoxydiphenylmethane* (**5**). White solid; TLC (ethyl acetate/hexane 1:1): R*_f_* = 0.90. ^1^H-NMR (300 MHz, CDCl_3_): δ 7.46–7.28 (m, 10H), 5.44 (s,1H), 3.61 (q, 2H, *J =*6.9 Hz), 1.35 (t, 3H, *J =*6.9 Hz). ^13^C-NMR (75 MHz, CDCl_3_): δ 142.3, 128.1, 127.1, 126.7, 83.2, 64.3, 15.1.

*Recovered Thymidine* (**1**). White solid; TLC (DCM/MeOH 9:1): R*_f_* = 0.40. ^1^H-NMR (300 MHz, CD_3_OD): δ 7.2 (bs, 1H, NH), 6.29 (t, 1H, H-1′, *J =* 6.6 Hz), 4.43–4.39 (m, 1H), 3.94–3.90 (m, 1H), 3.84–3.71 (m, 2H), 2.27–2.22 (m, 2H), 1.89 (s, 3H, CH_3_). ^13^C-NMR (75 MHz, CD_3_OD): δ 164.7, 150.6, 136.5, 109.8, 87.1, 84.5, 70.5, 61.1, 39.5, 10.7.

## 4. Conclusions

A convenient and effective method for the protection of hydroxyl groups of nucleosides as the corresponding diphenylmethyl ether has been developed with the aid of a transition metal catalyst. In addition, only by changing the solvent, the corresponding diphenylmethyl ethers can be deprotected under similar conditions using palladium (II) chloride as catalyst. With both protection and deprotection reactions proceeding under mild conditions and with nice selectivity, this high yielding strategy can be useful for the synthesis of complex nucleosides and nucleotides.

## Supplementary Materials

A supplementary file with scanned spectra is provided. Supplementary materials can be accessed at: http://www.mdpi.com/1420-3049/18/7/8524/s1.
