# Supplementary material for: A Straightforward Diphenylmethyl Protection Method and Deprotection of Some Pyrimidine Nucleosides
Source: Molecules. 2013 Jul 18;18(7):8524–34. doi: 10.3390/molecules18078524 (PMC6270414; doi:10.3390/molecules18078524)

Supporting Materials

^{1}H and ^{13}C-NMR Spectra

Figure S1. ^{1}H and ^{13}C-NMR Spectra of 5′-*O*-benzhydryl-thymidine (**3a**).

HRMS (ES+)

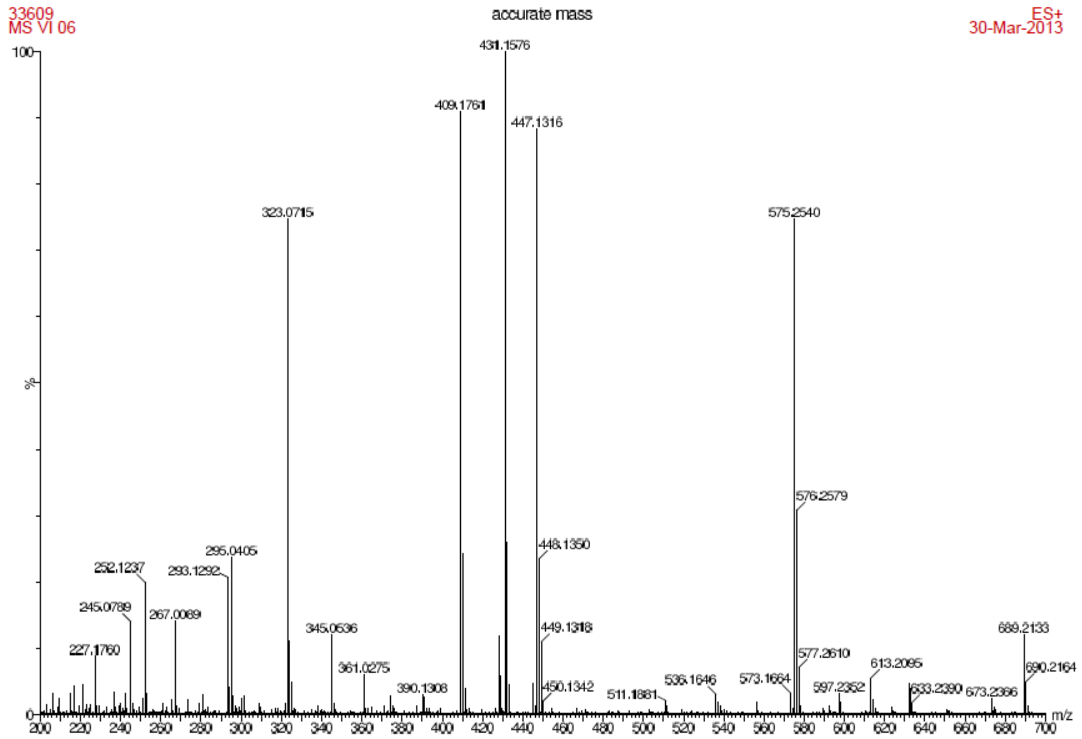

^{1}H-NMR (300 MHz, $CDCl_3$)

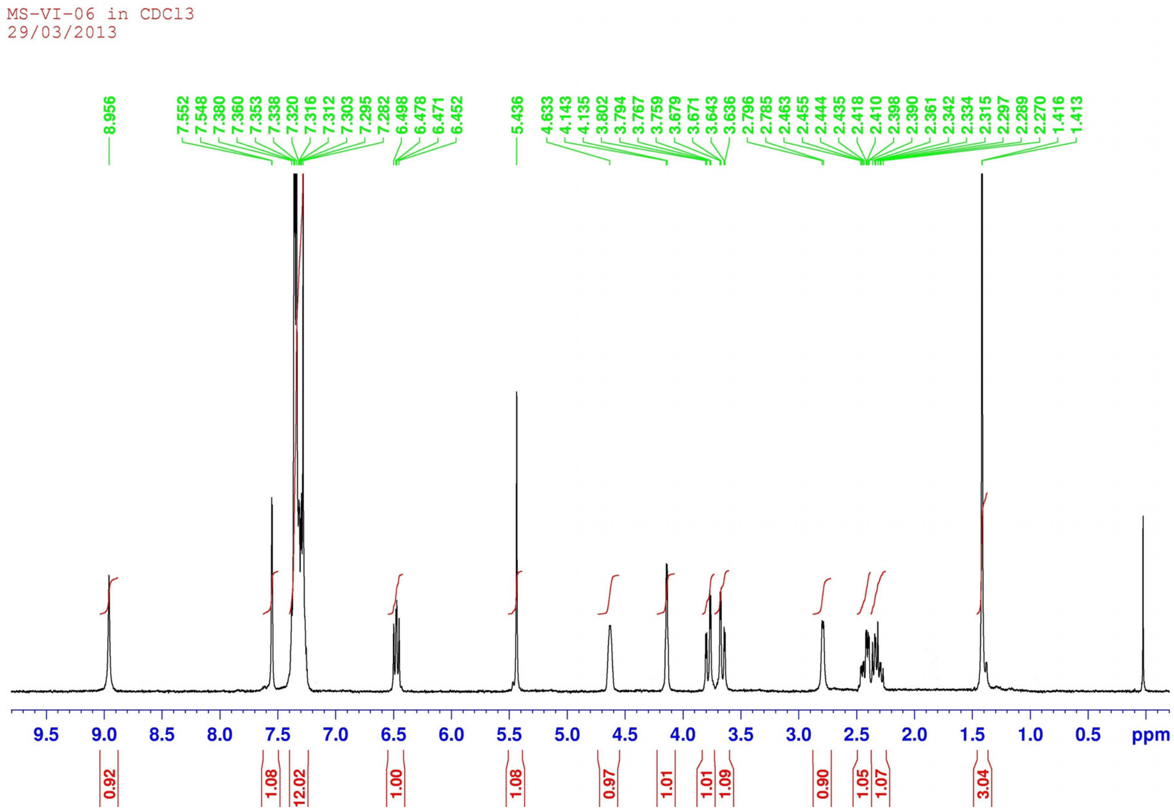

^{13}C-NMR (75 MHz, $CDCl_3$)

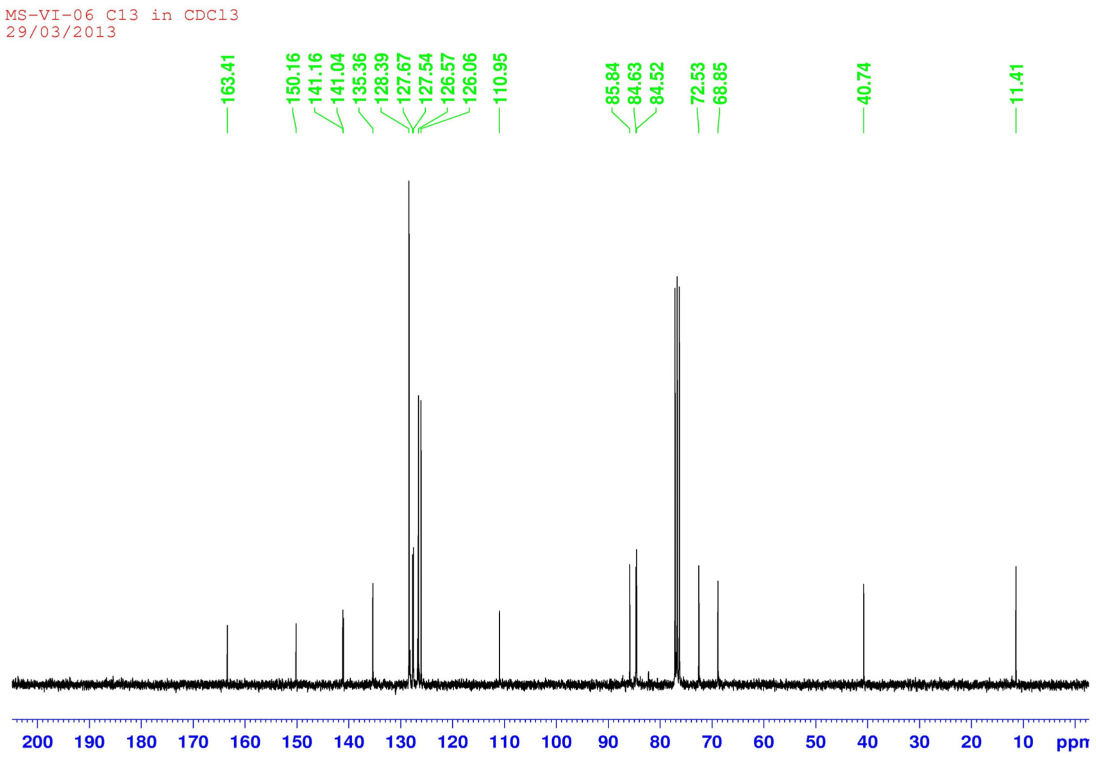

Figure S2. ^{1}H and ^{13}C-NMR Spectra of 3′,5′-di-*O*-benzhydryl-thymidine (**3b**).

HRMS (ES+)

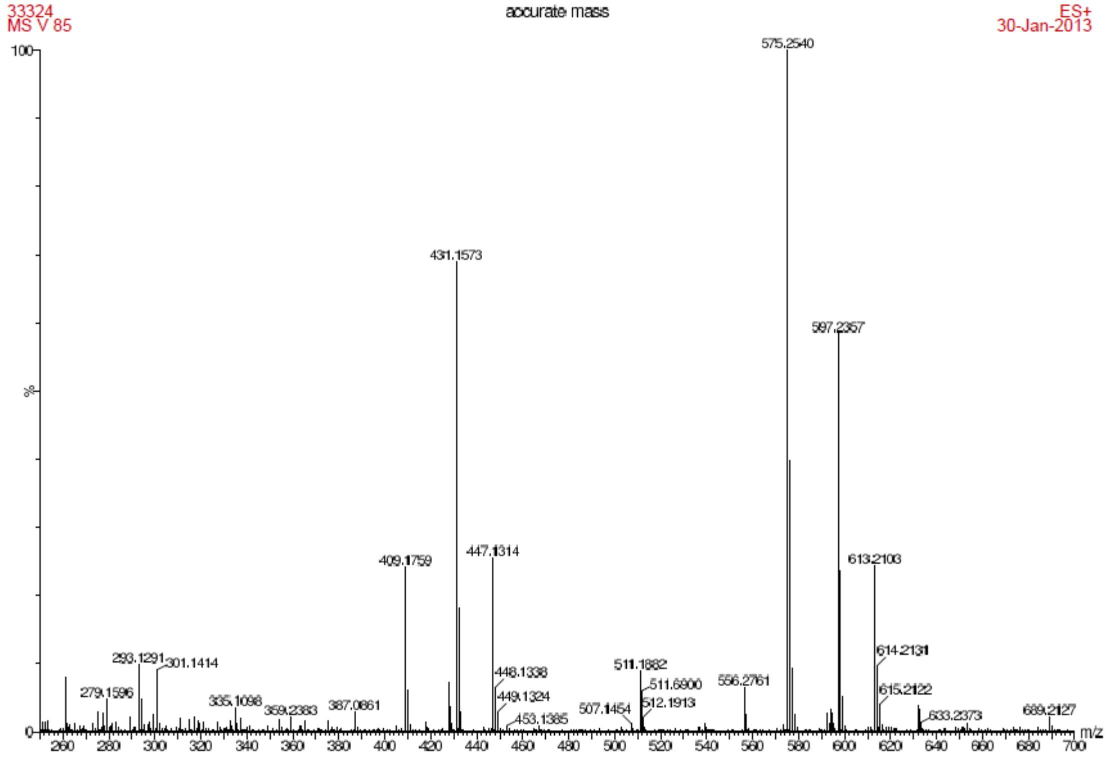

^{1}H-NMR (300 MHz, $CDCl_3$)

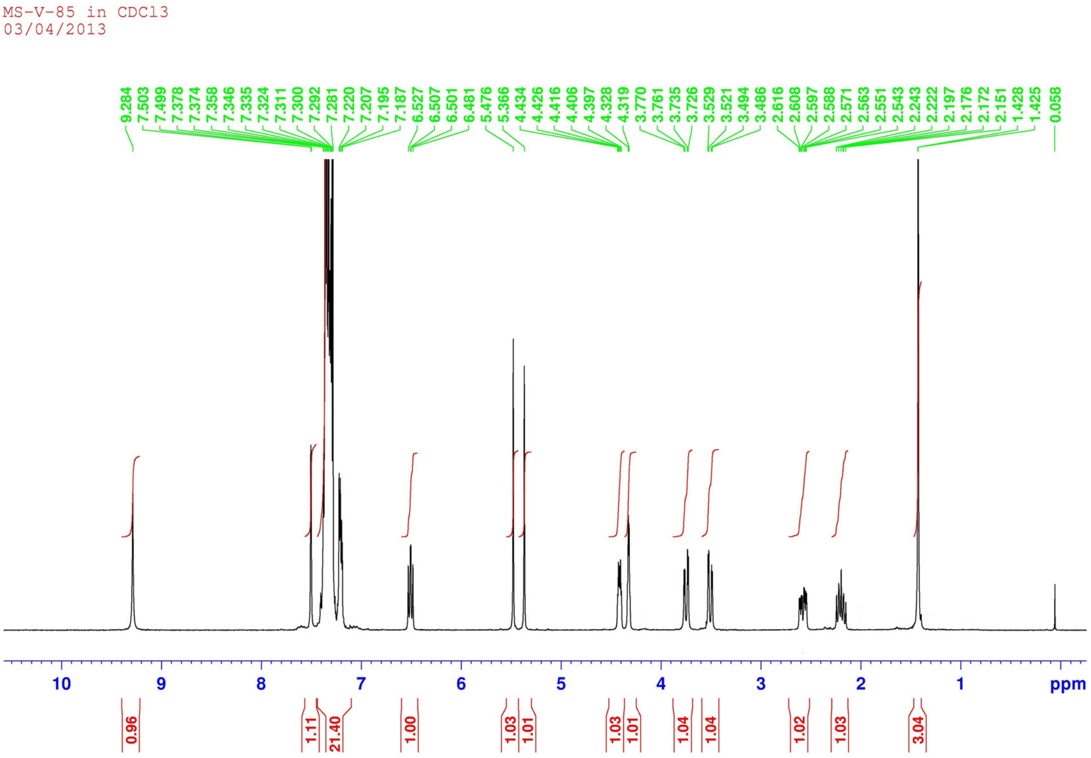

^{13}C-NMR (75 MHz, $CDCl_3$)

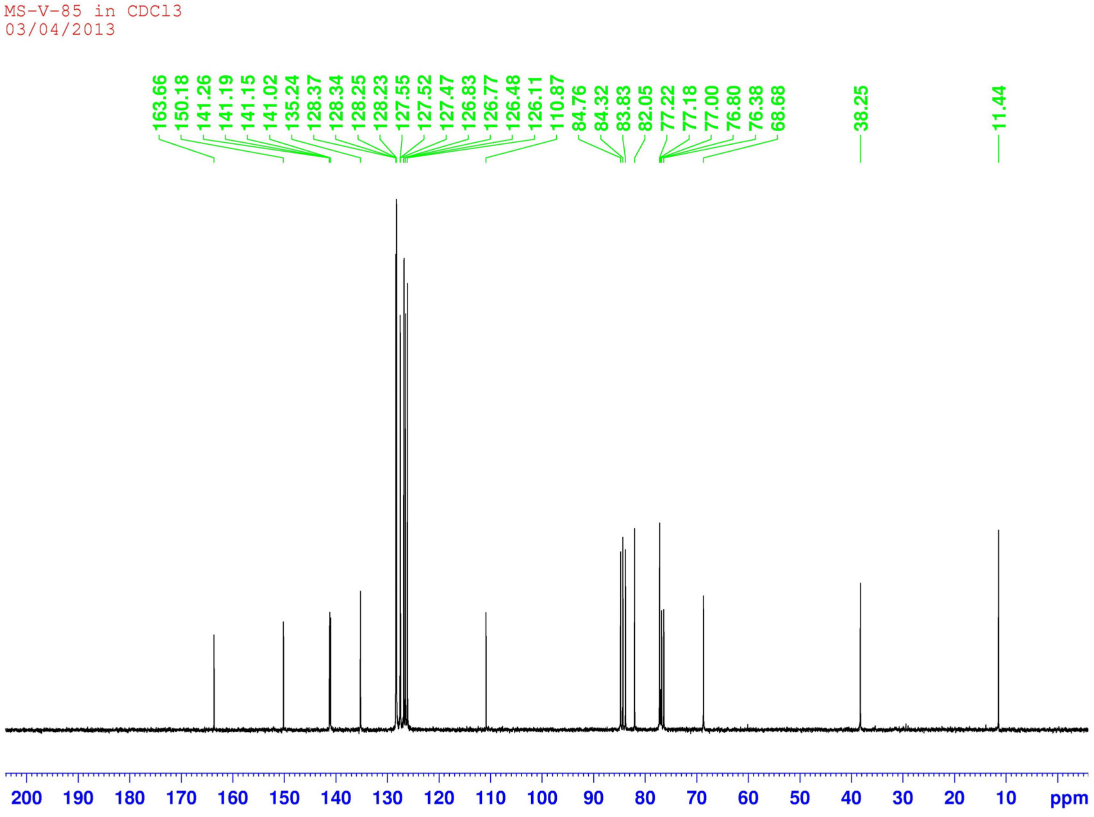

Figure S3. ¹H and ¹³C-NMR Spectra of 5′-*O*-benzhydryl-2′-deoxyuridine (**6a**).

HRMS (ES+)

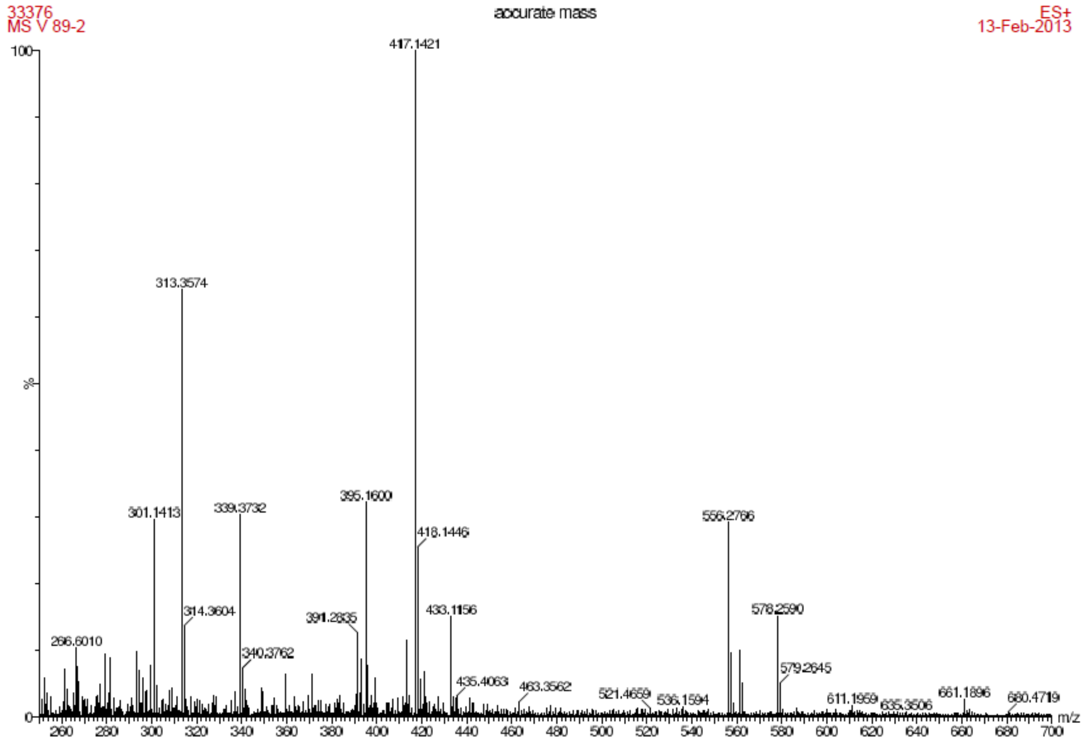

^{1}H-NMR (600 MHz, $CDCl_3$)

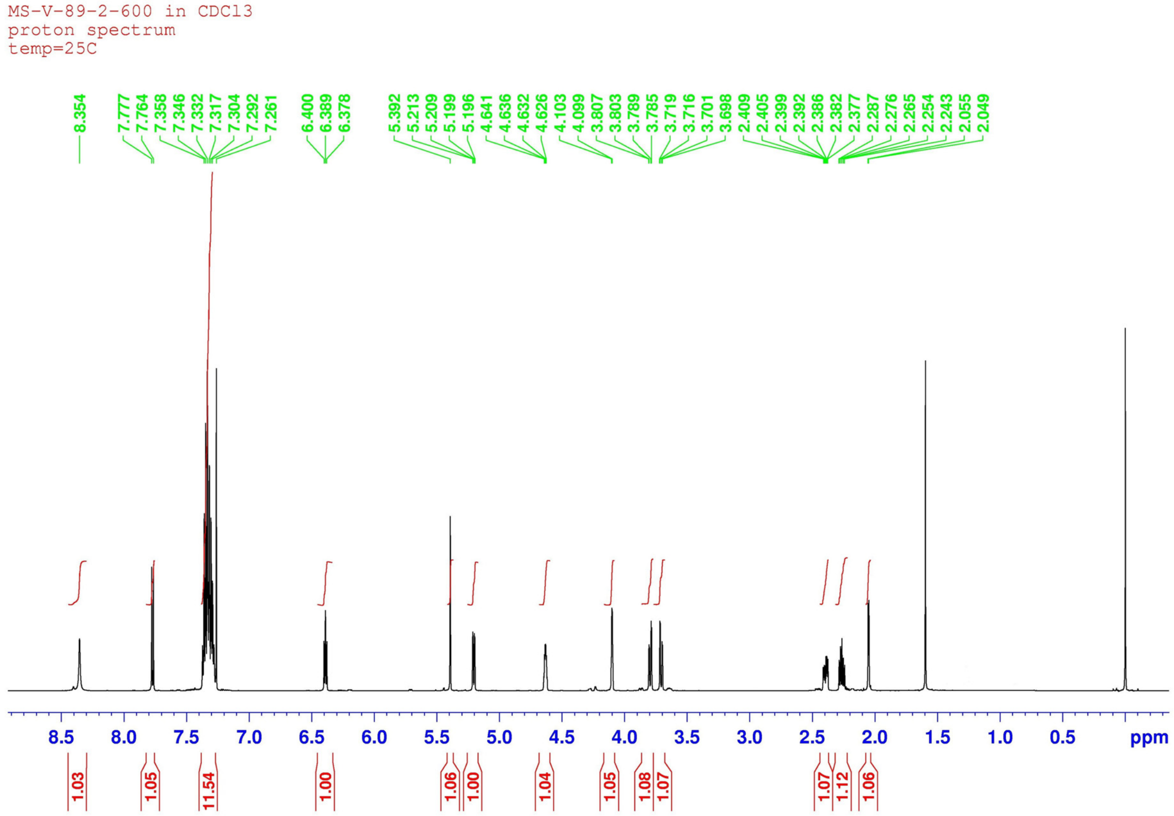

^{13}C-NMR (150 MHz, $CDCl_3$)

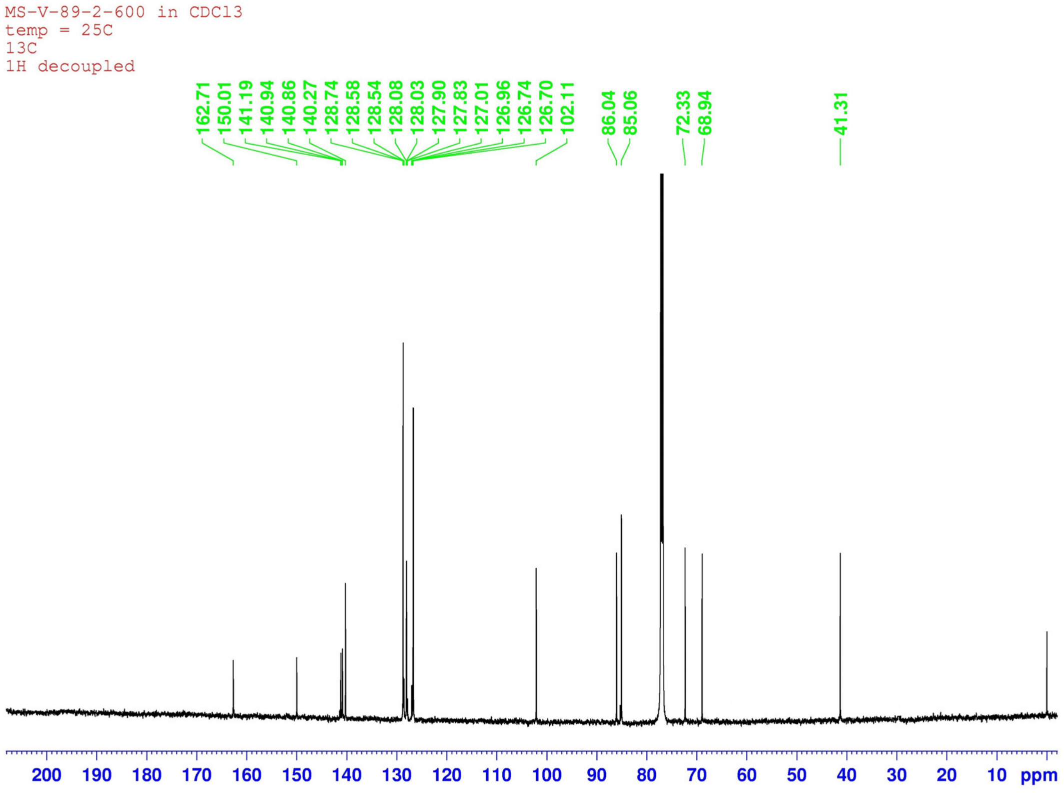

Figure S3. ^{1}H and ^{13}C-NMR Spectra of 3′,5′-di-*O*-benzhydryl-2′-deoxyuridine (**6b**).

HRMS (ES+)

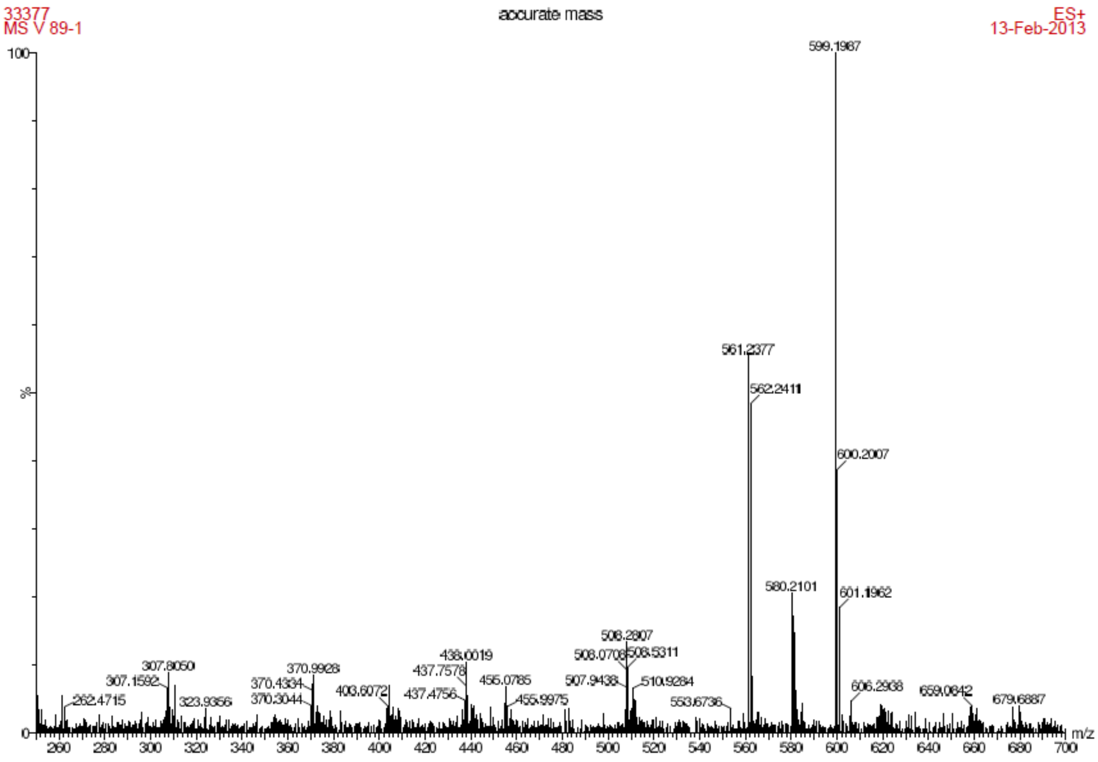

^{1}H-NMR (600 MHz, $CDCl_3$)

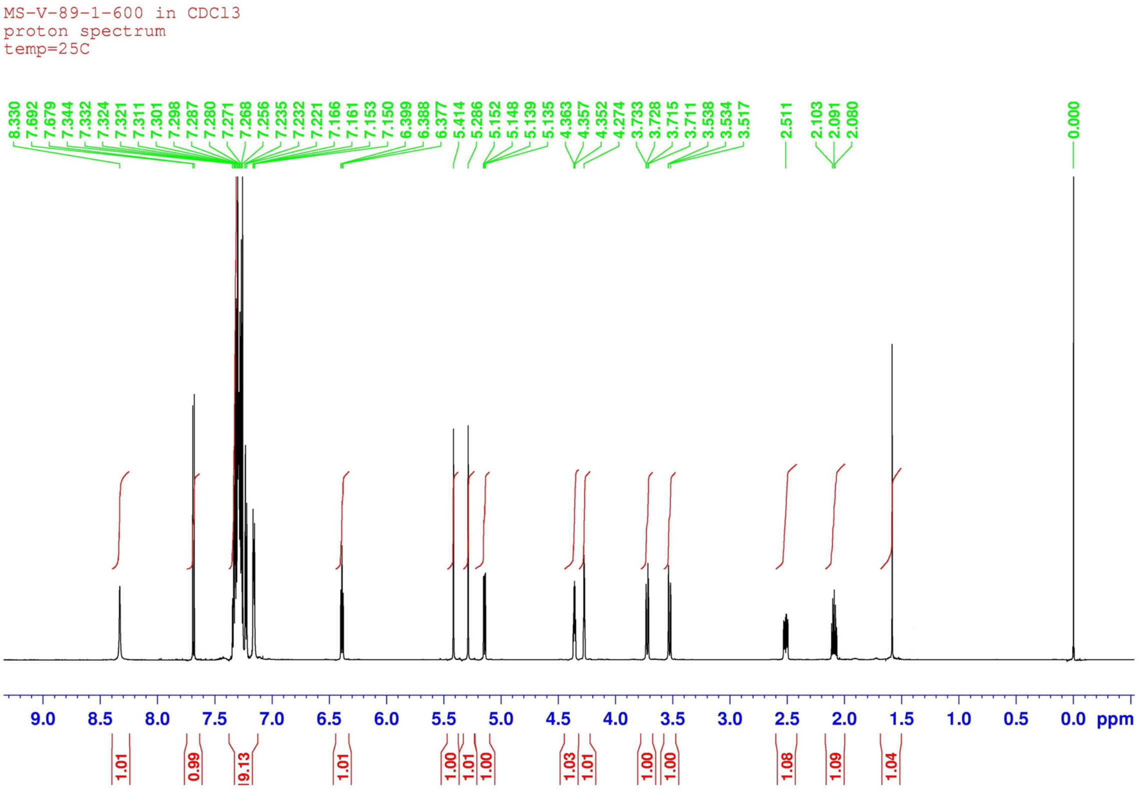

^{13}C-NMR (150 MHz, $CDCl_3$)

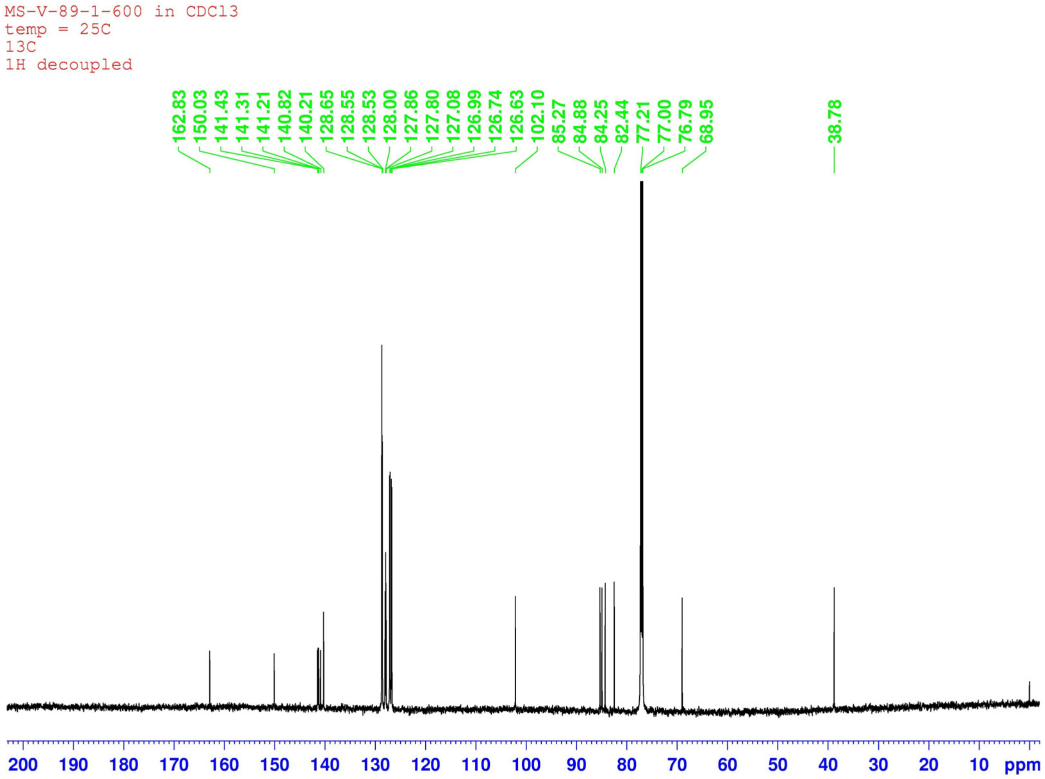

Figure S4. ^{1}H and ^{13}C-NMR Spectra of 5′-*O*-benzhydryl-5-fluoro-2′-deoxyuridine (**7a**)

HRMS (ES+)

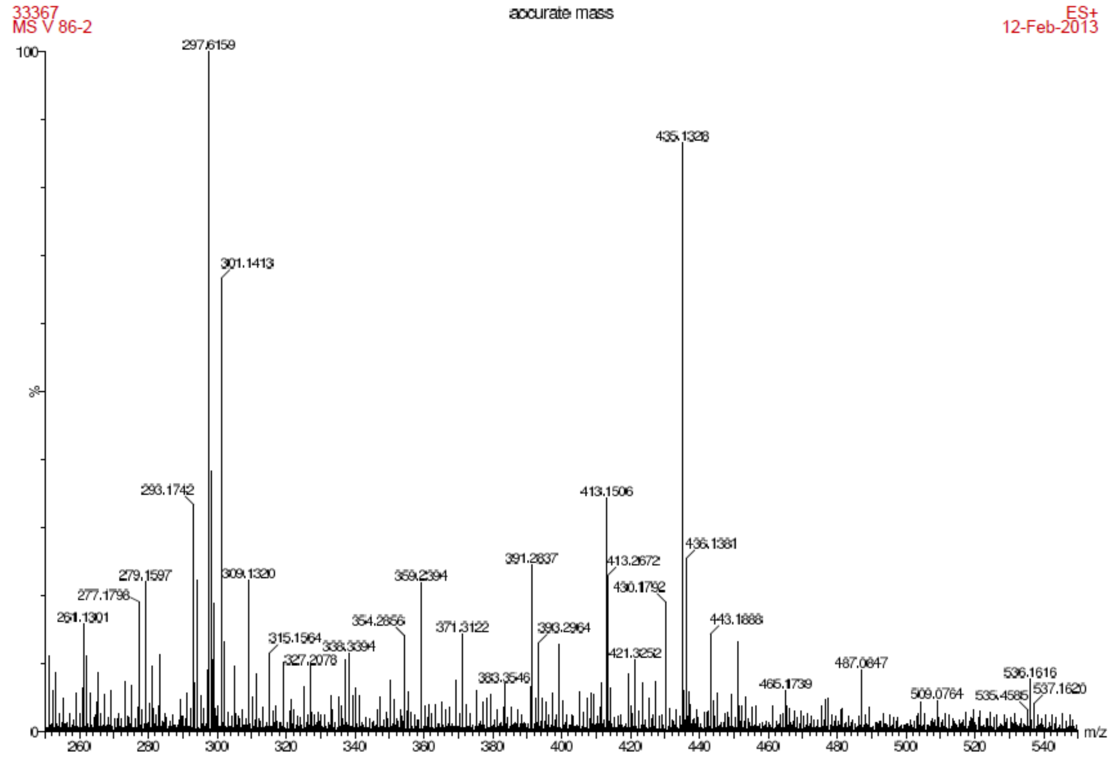

^{1}H-NMR (600 MHz, $CDCl_3$)

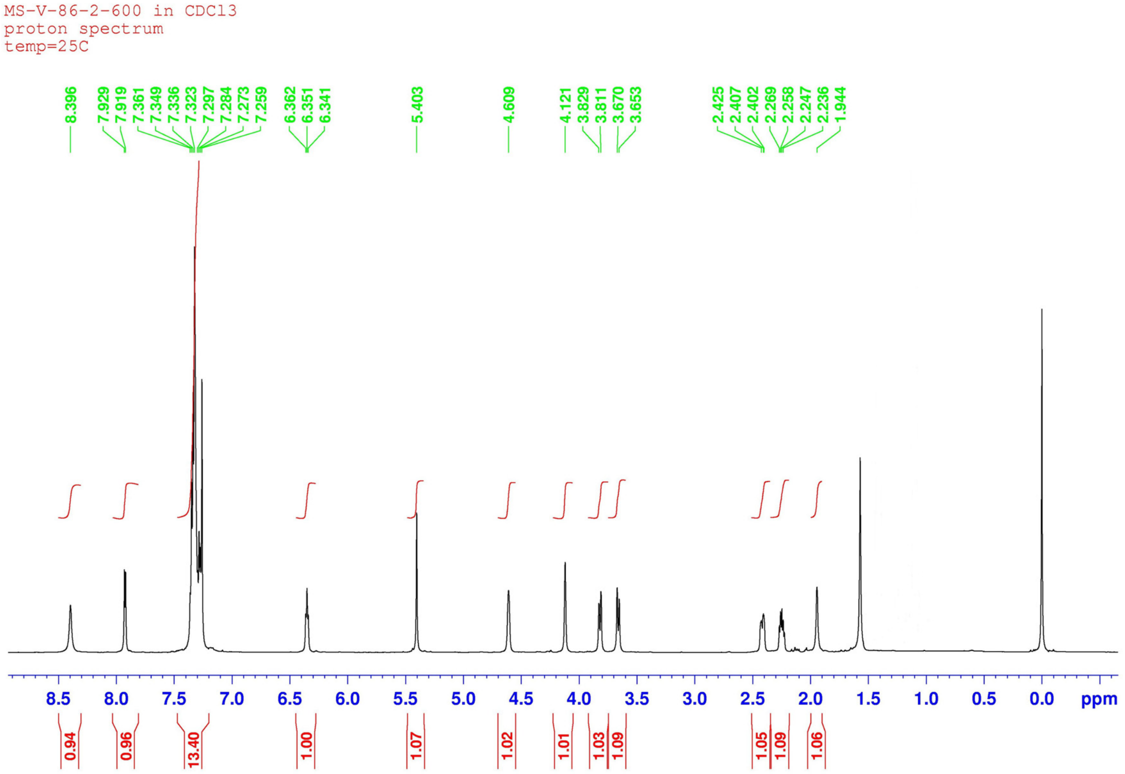

^{13}C-NMR (150 MHz, $CDCl_3$)

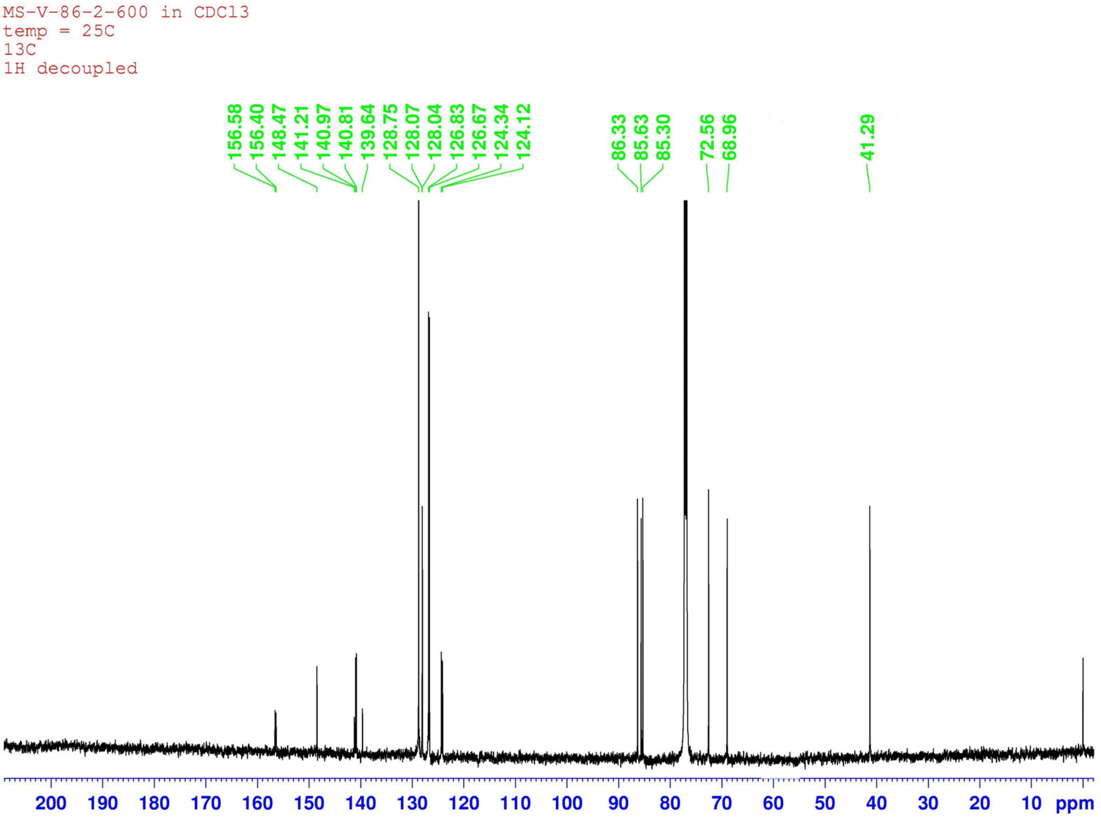

Figure S5. ^{1}H and ^{13}C-NMR Spectra of 3′,5′-di-*O*-benzhydry-5-fluoro-2′-deoxyuridine (**7b**).

HRMS (ES+)

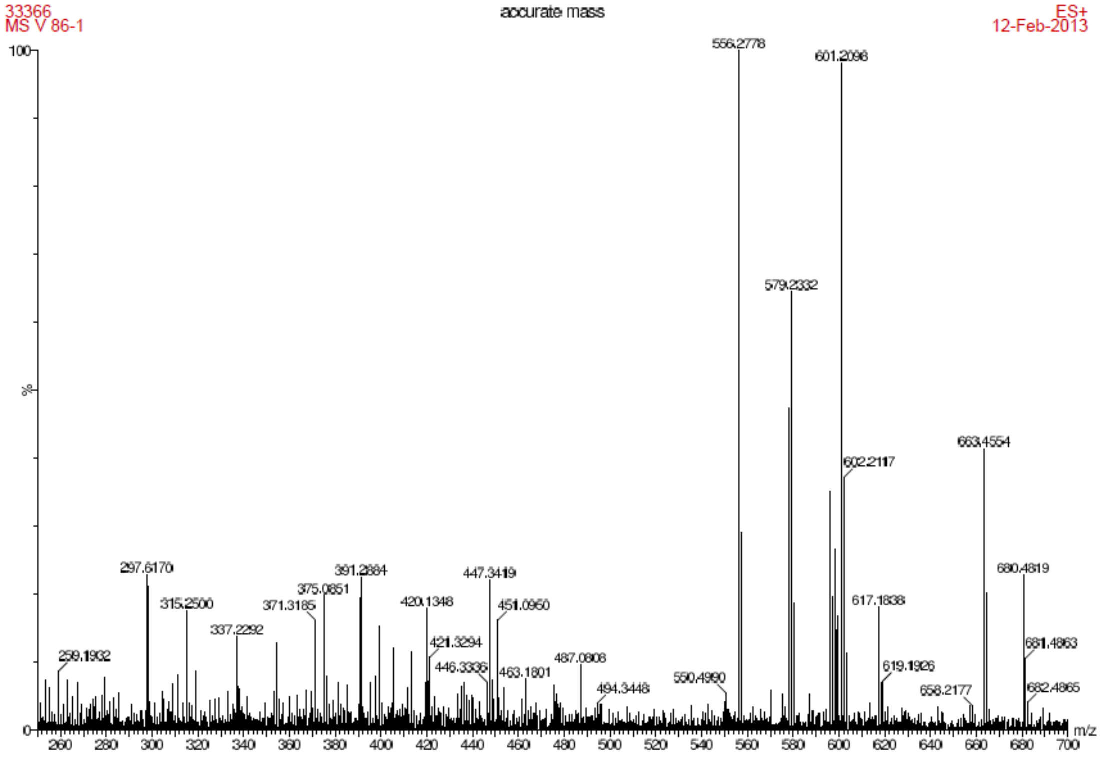

^{1}H-NMR (600 MHz,$CDCl_3$)

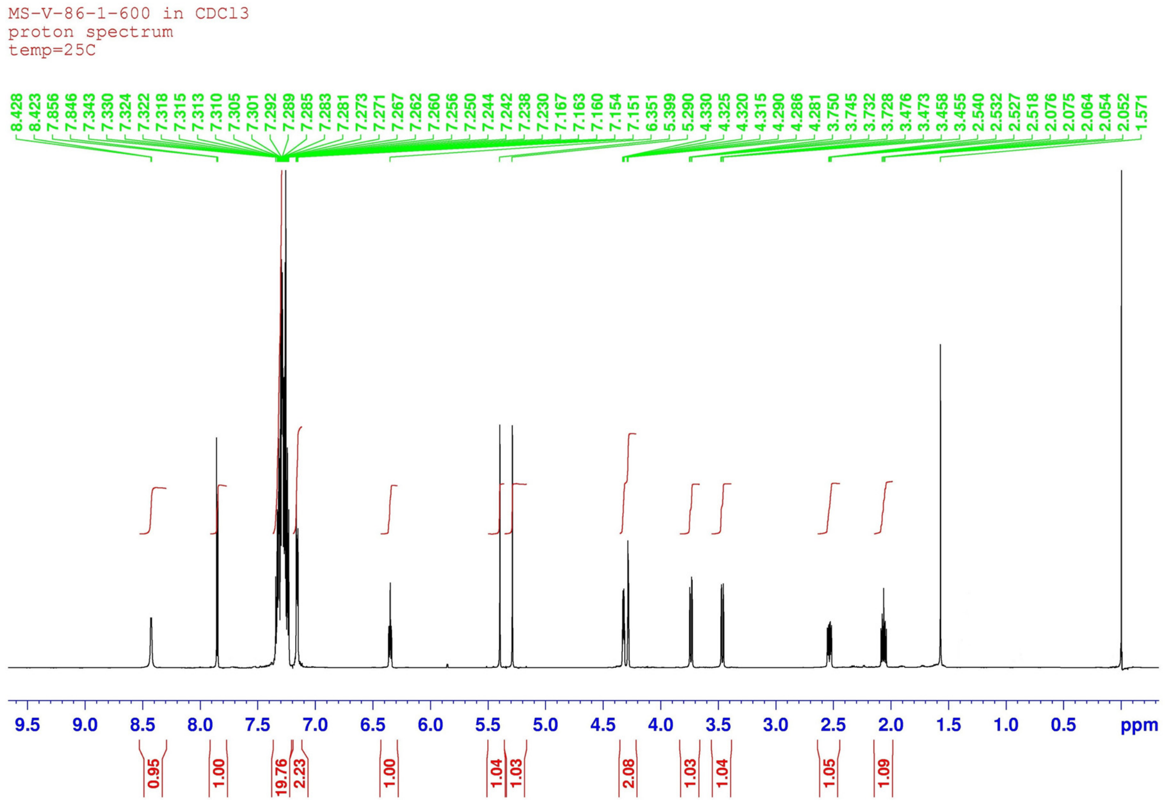

^{13}C-NMR (150 MHz, $CDCl_3$)

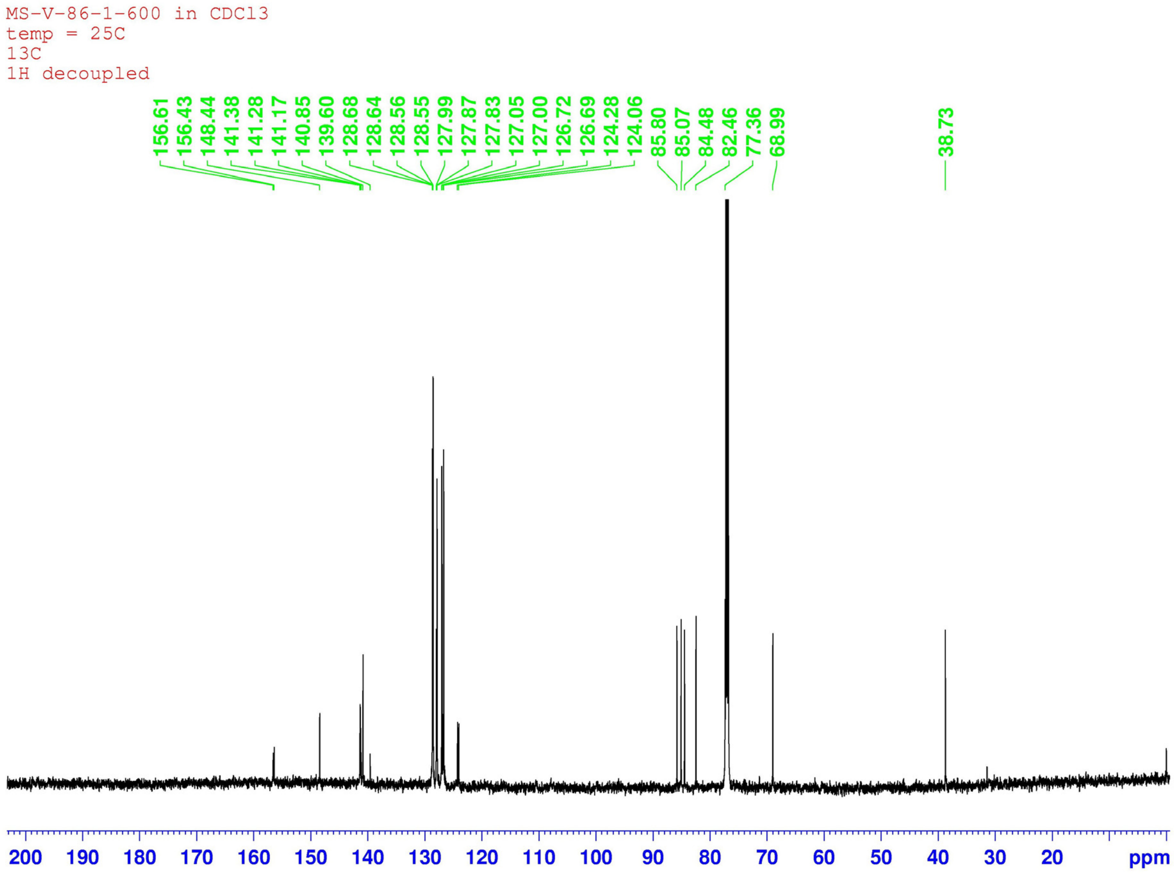

Figure S6. ^{1}H and ^{13}C-NMR Spectra of 2′,5′-di-*O*-benzhydryl-uridine (**8b**).

HRMS (ES+)

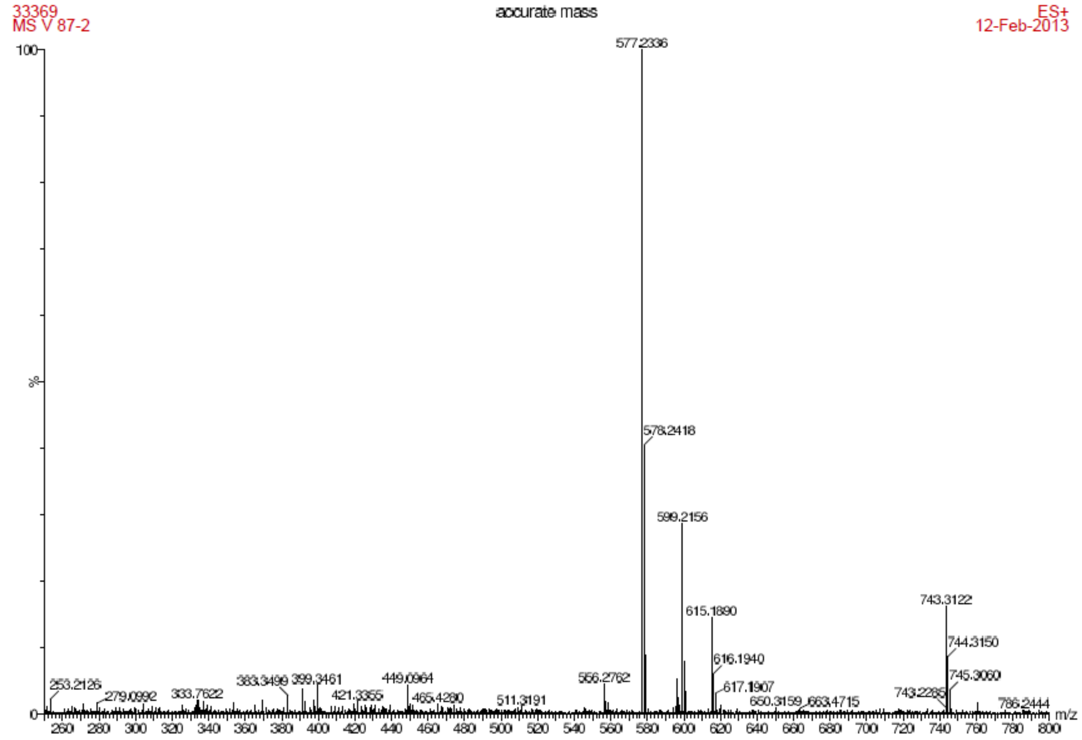

^{1}H-NMR (600 MHz, $CDCl_3$)

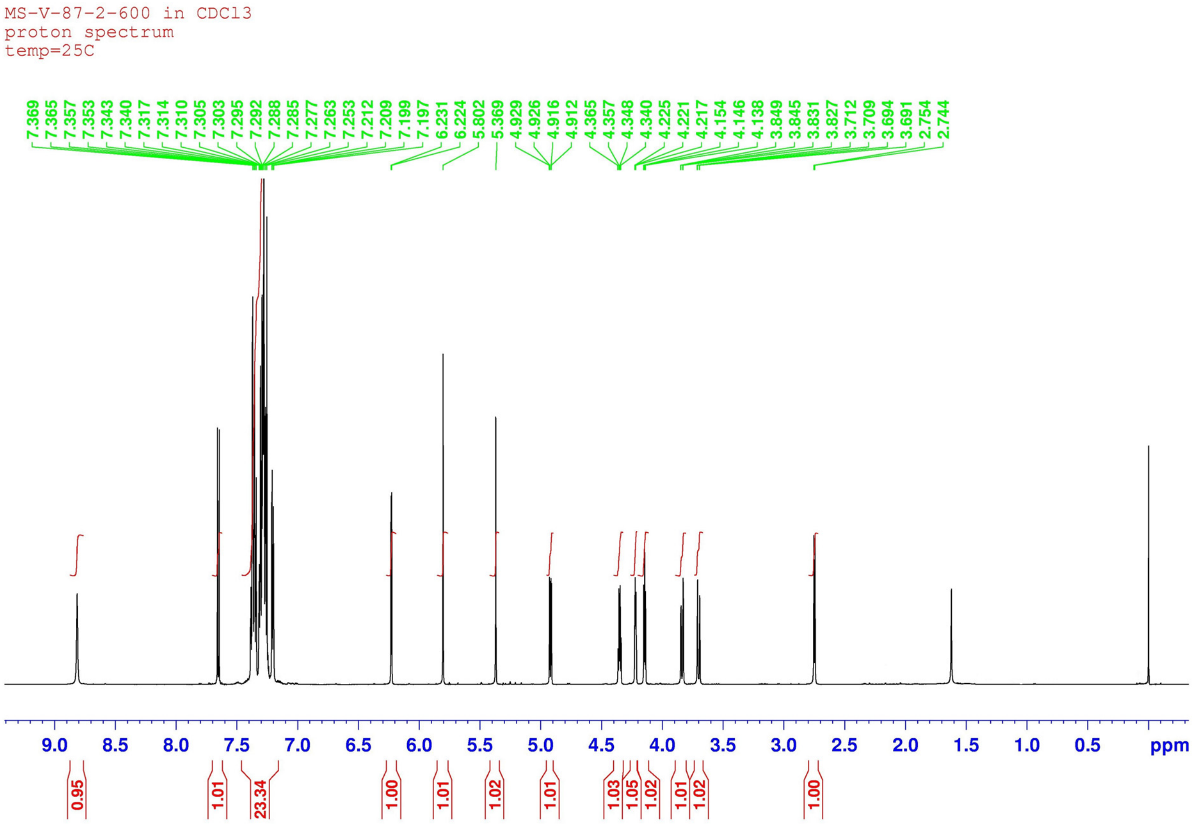

^{13}C-NMR (150 MHz, $CDCl_3$)

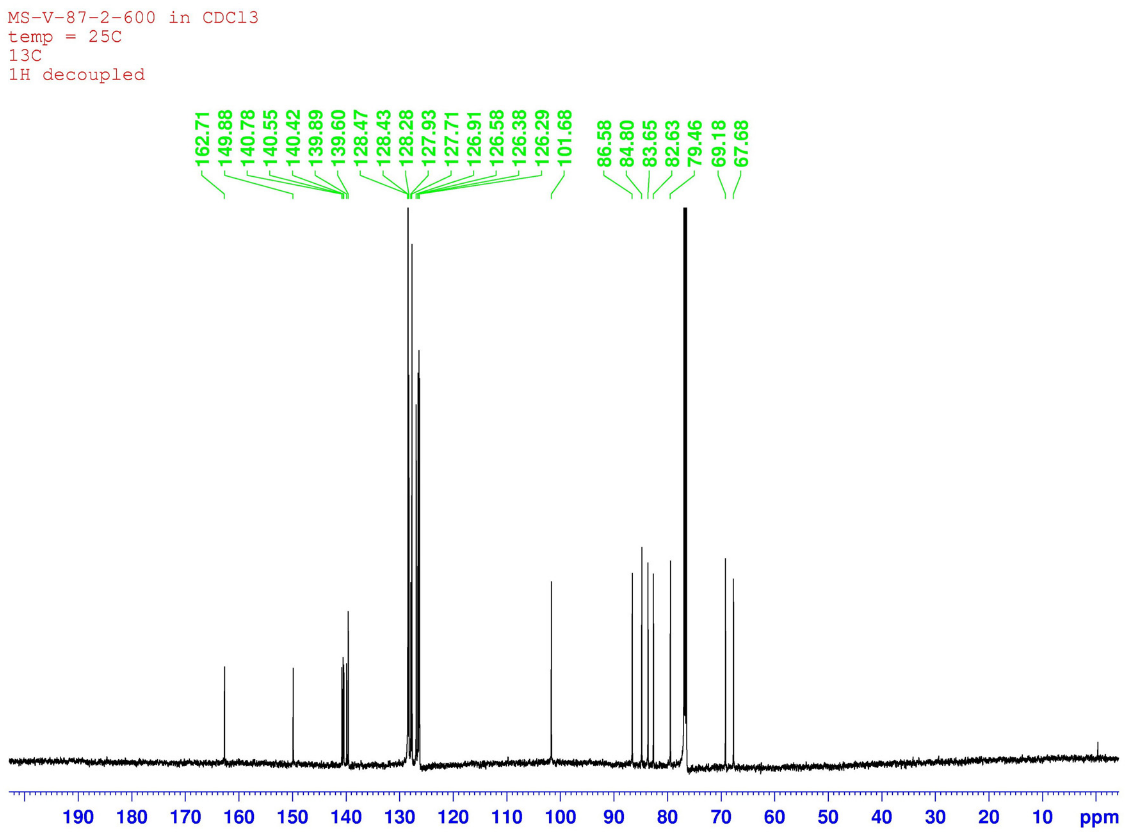

Figure S7. ^{1}H and ^{13}C-NMR Spectra of 3′,5′-di-*O*-benzhydryl-uridine (**8c**)

HRMS (ES+)

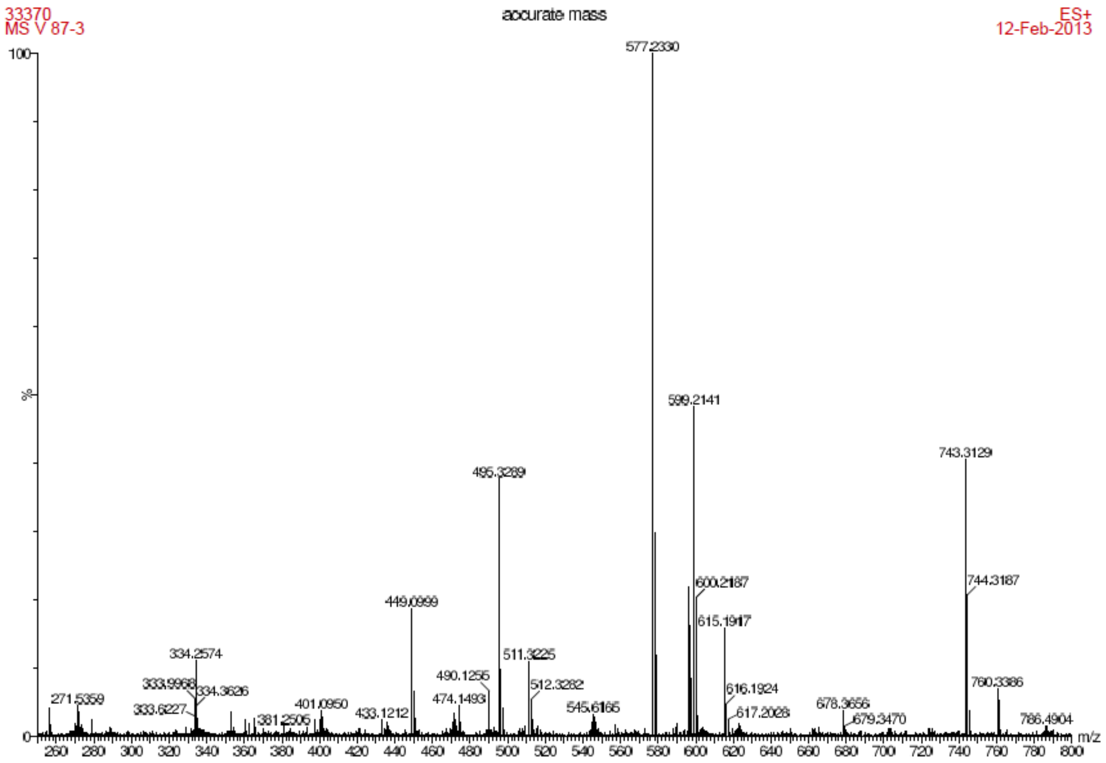

^{1}H-NMR (600 MHz, $CDCl_3$)

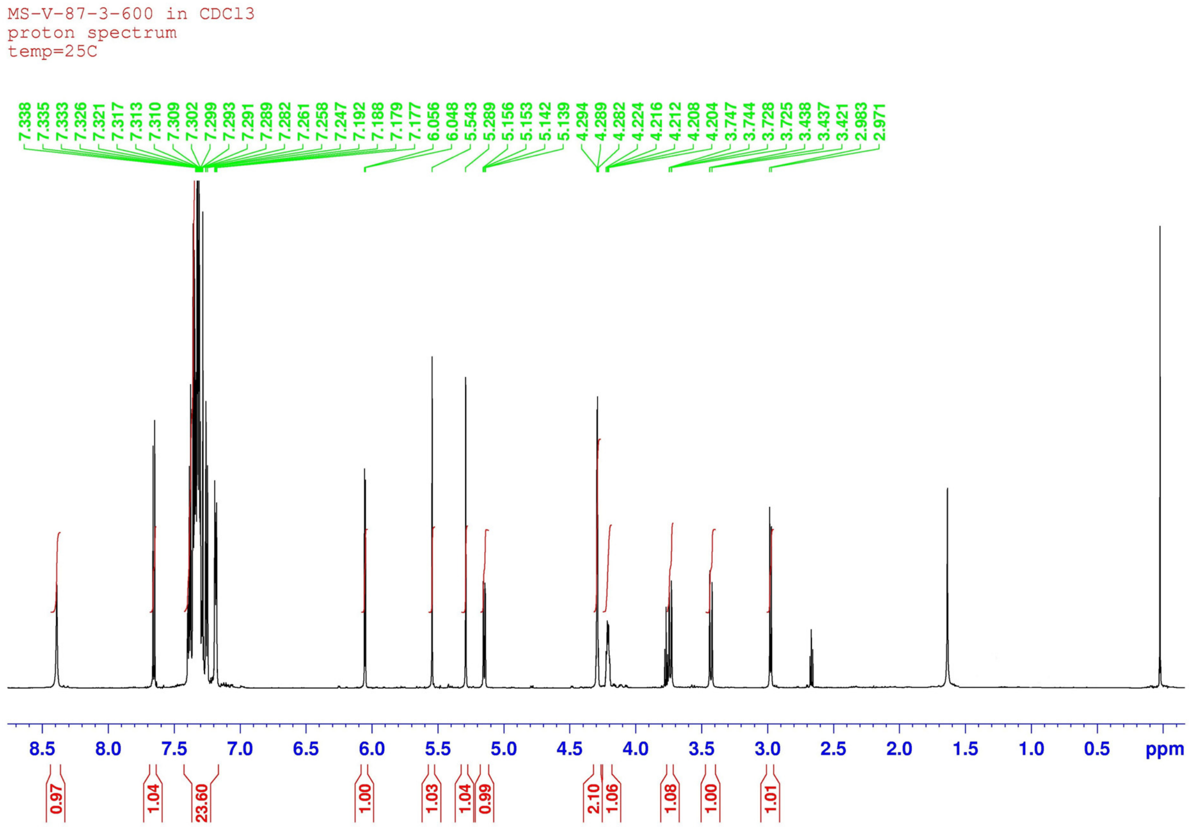

^{13}C-NMR (150 MHz, $CDCl_3$)

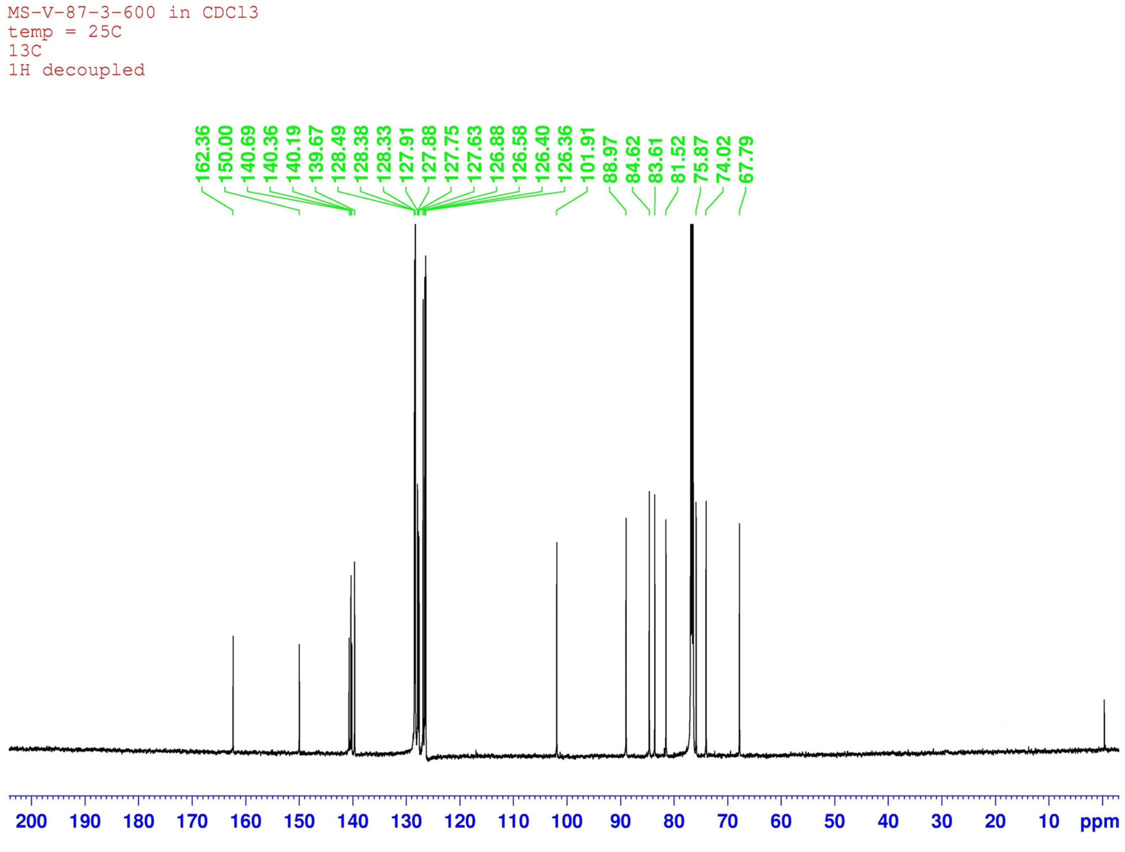

Figure S7. ^{1}H and ^{13}C-NMR Spectra of 2′,3′,5′-tri-*O*-benzhydryl-uridine (**8d**).

HRMS (ES+)

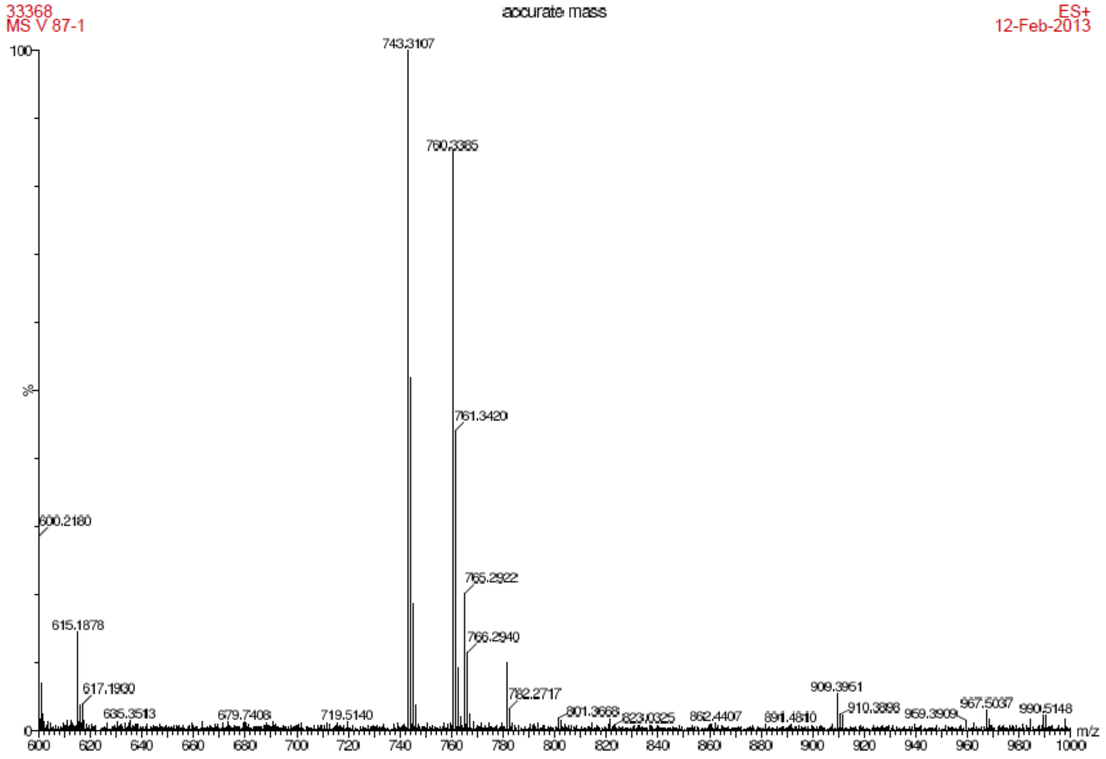

^{1}H-NMR (600 MHz, $CDCl_3$)

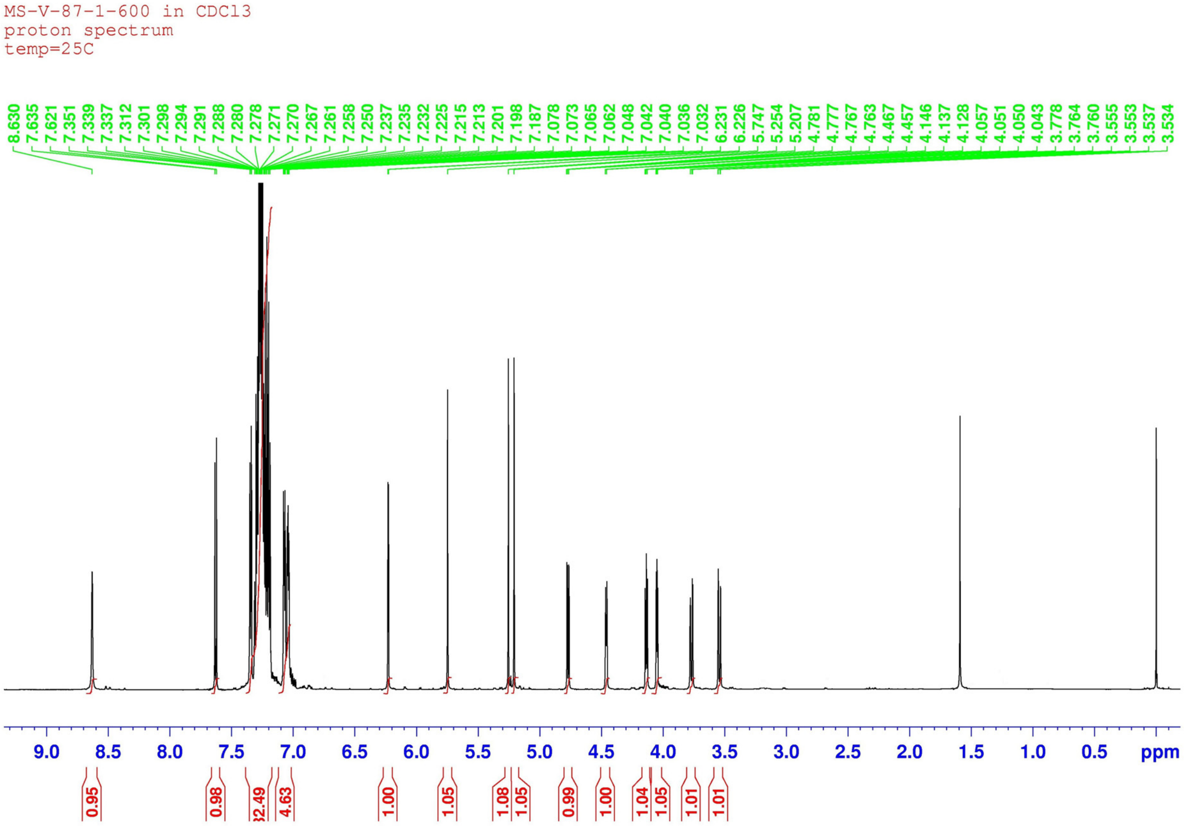

^{13}C-NMR (150 MHz, $CDCl_3$)

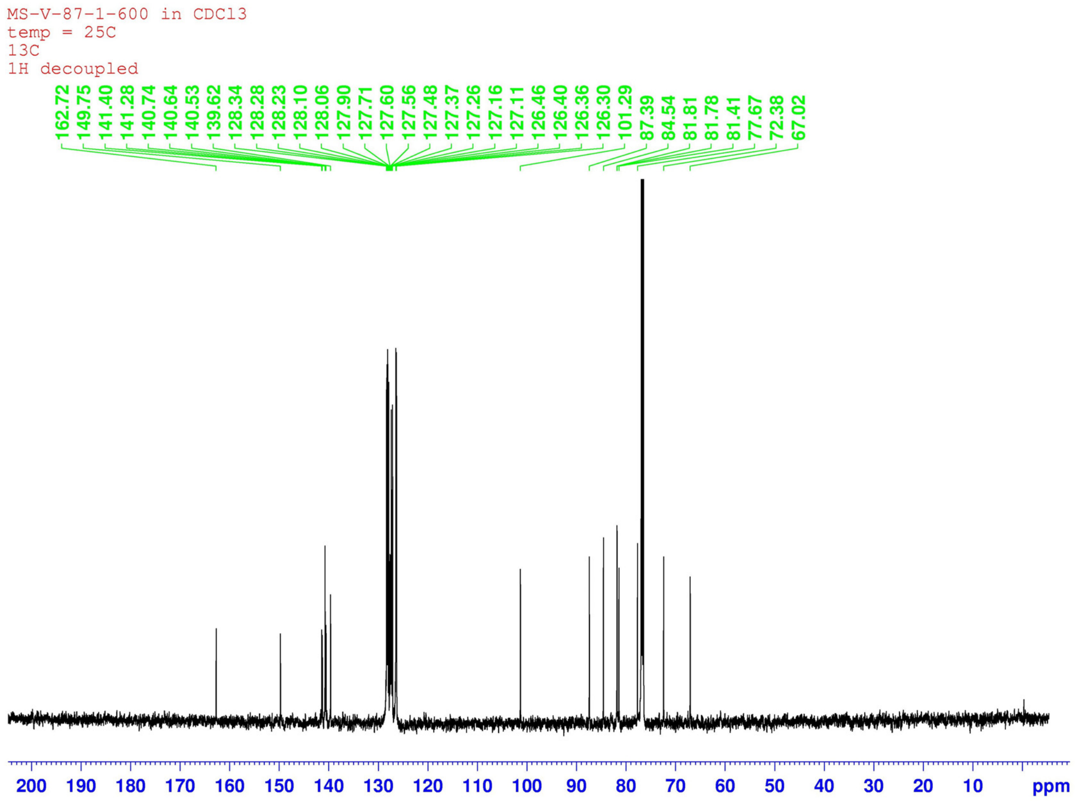

Figure S8. ^{1}H and ^{13}C-NMR Spectra of 2′,5′-di-*O*-benzhydryl-5-fluorouridine (**9b**).

HRMS (ES+)

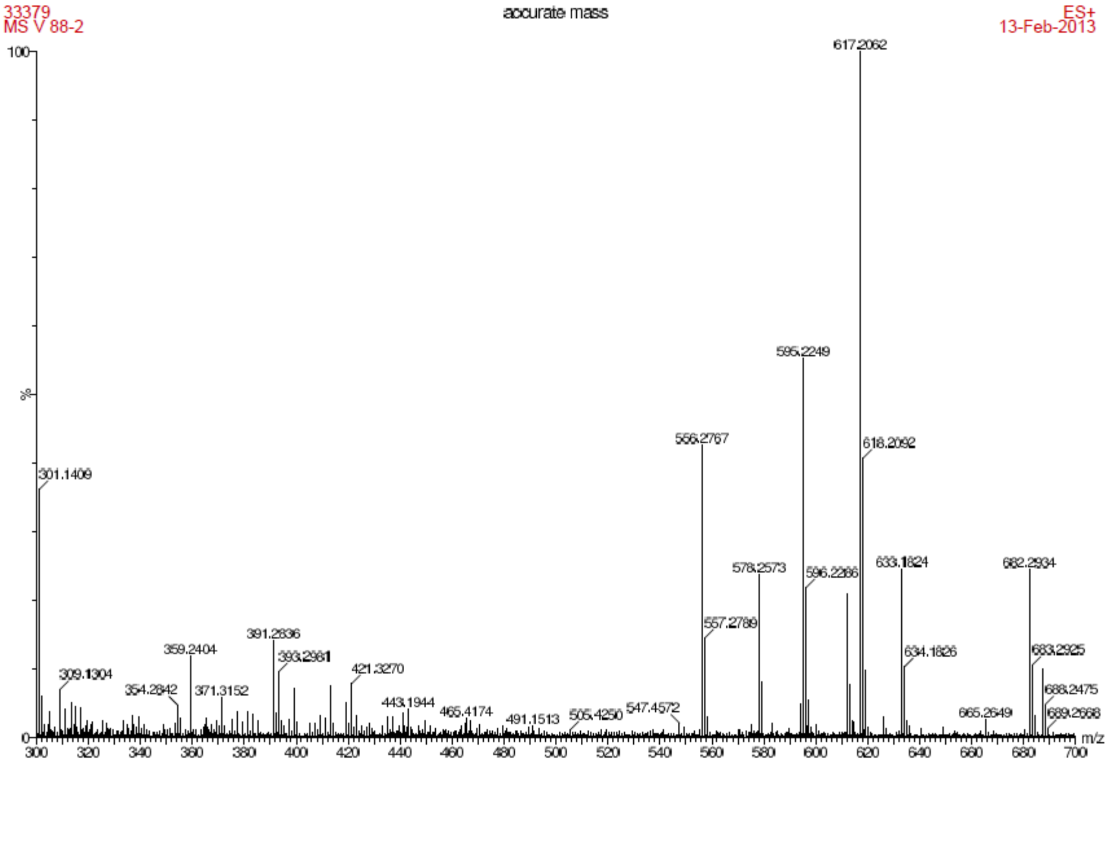

^{1}H-NMR (600 MHz, $CDCl_3$)

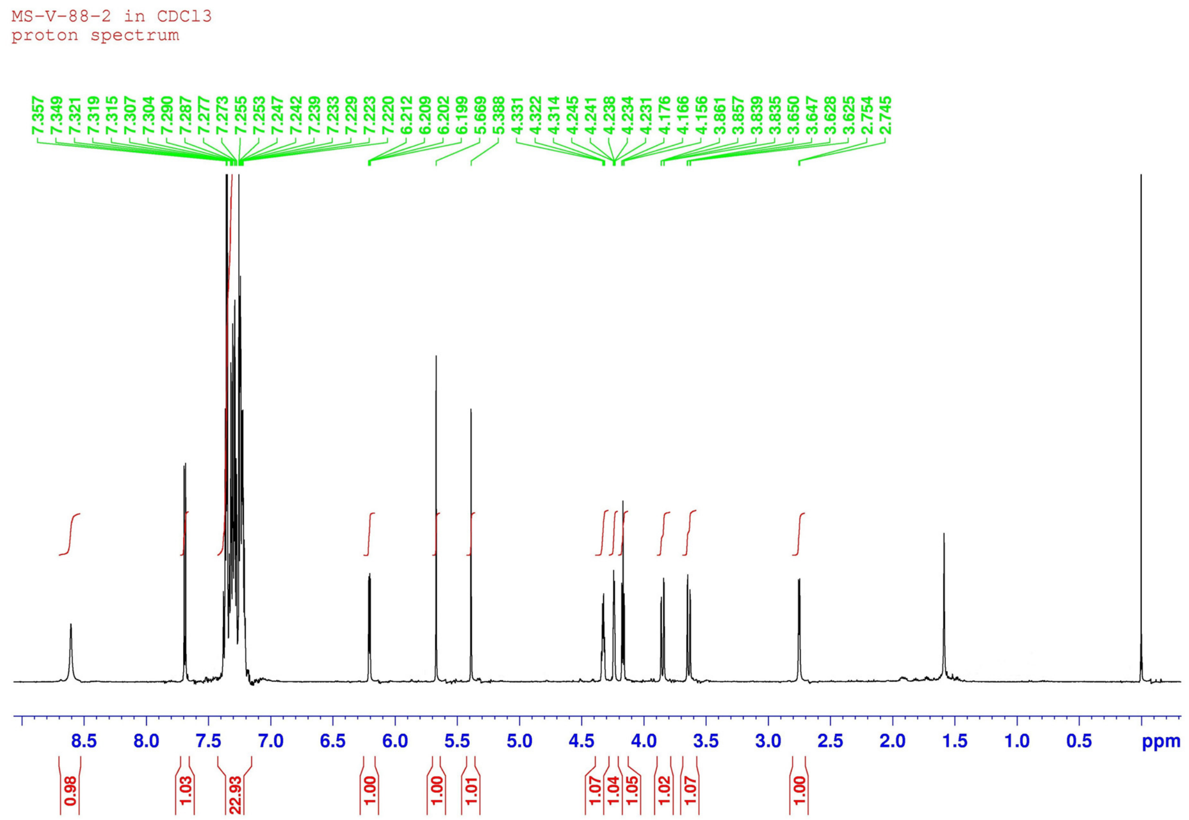

^{13}C-NMR (150 MHz, $CDCl_3$)

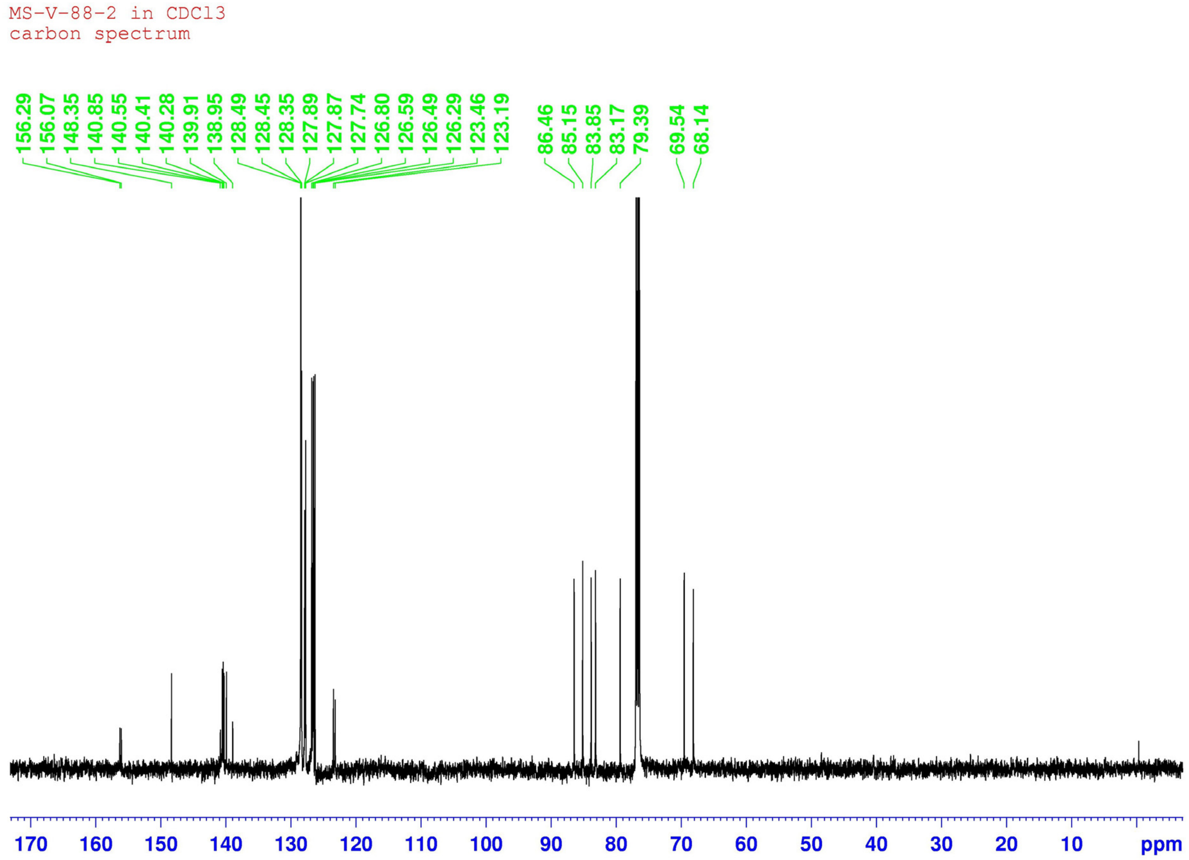

Figure S9. ^{1}H and ^{13}C-NMR Spectra of 3′,5′-tri-*O*-benzhydryl-5-fluorouridine (**9c**).

HRMS (ES+)

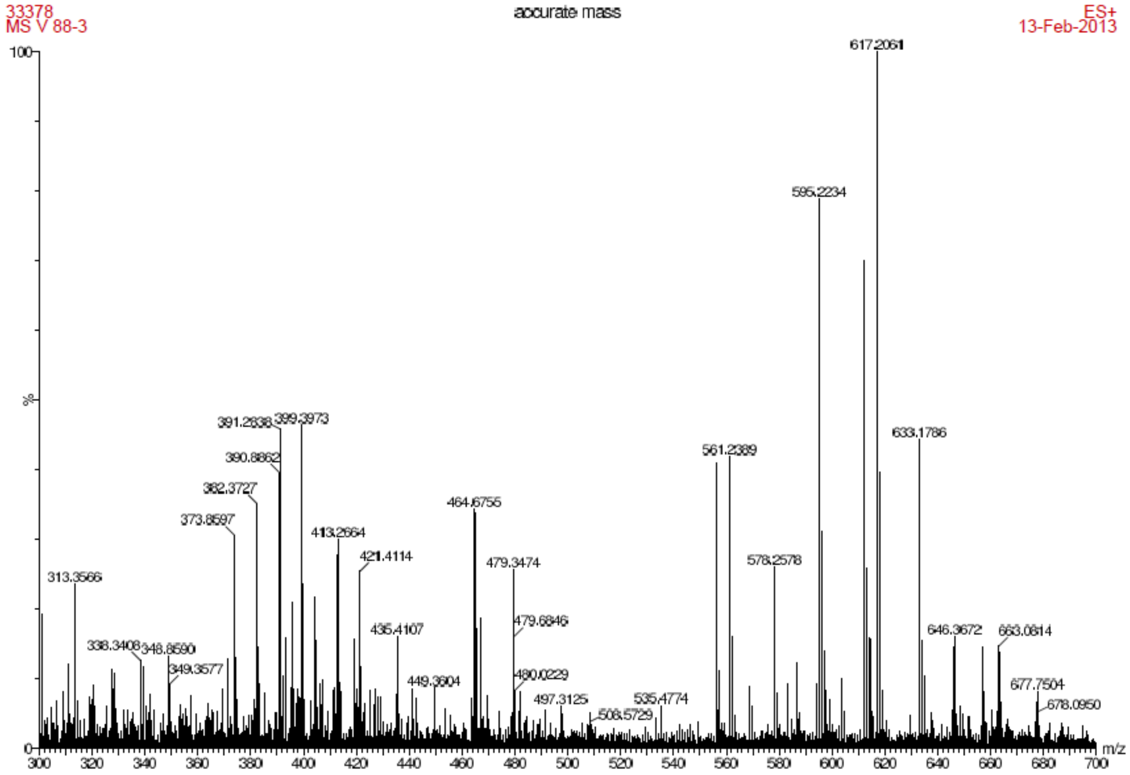

^{1}H-NMR (600 MHz, $CDCl_3$)

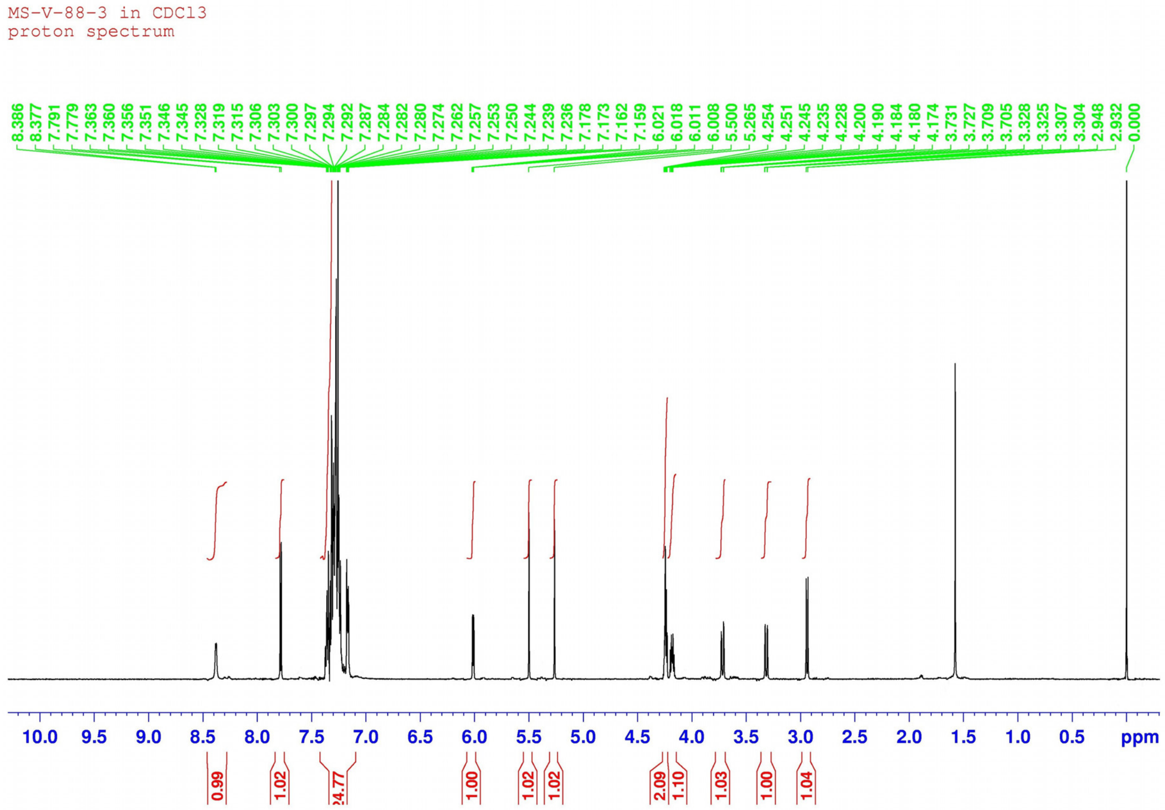

^{13}C-NMR (150 MHz, $CDCl_3$)

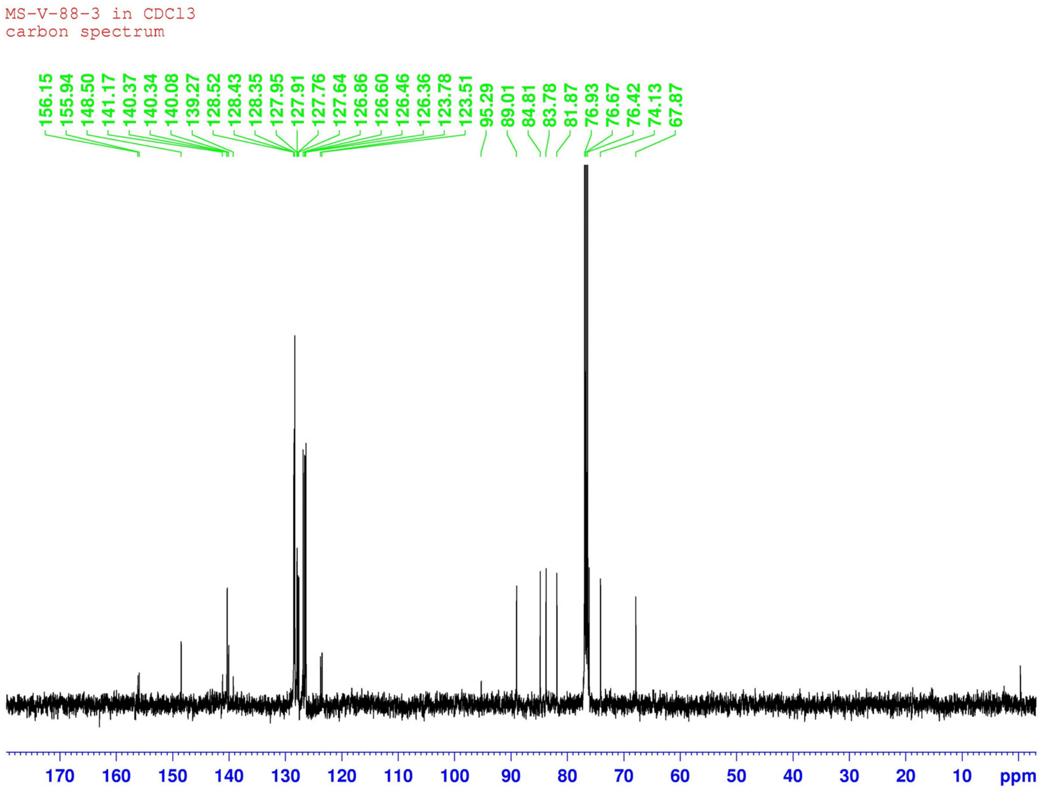

Figure S10. ^{1}H and ^{13}C-NMR Spectra of 2′,3′,5′-tri-*O*-benzhydryl-5-fluorouridine (**9d**).

HRMS (ES+)

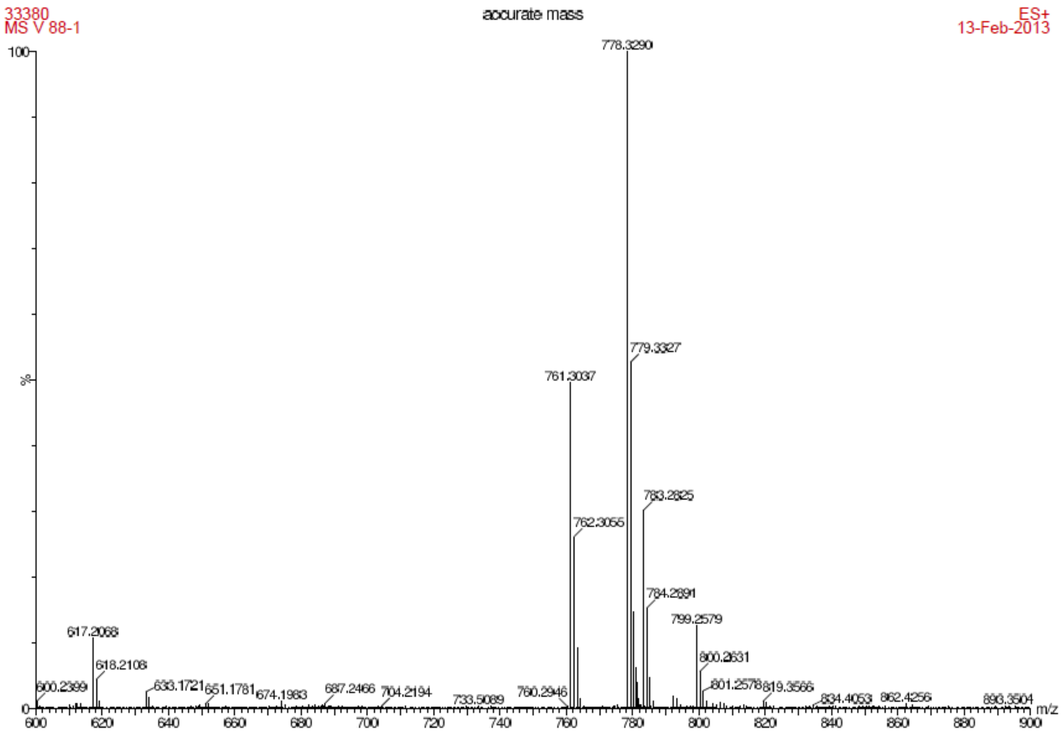

^{1}H-NMR (600 MHz, $CDCl_3$)

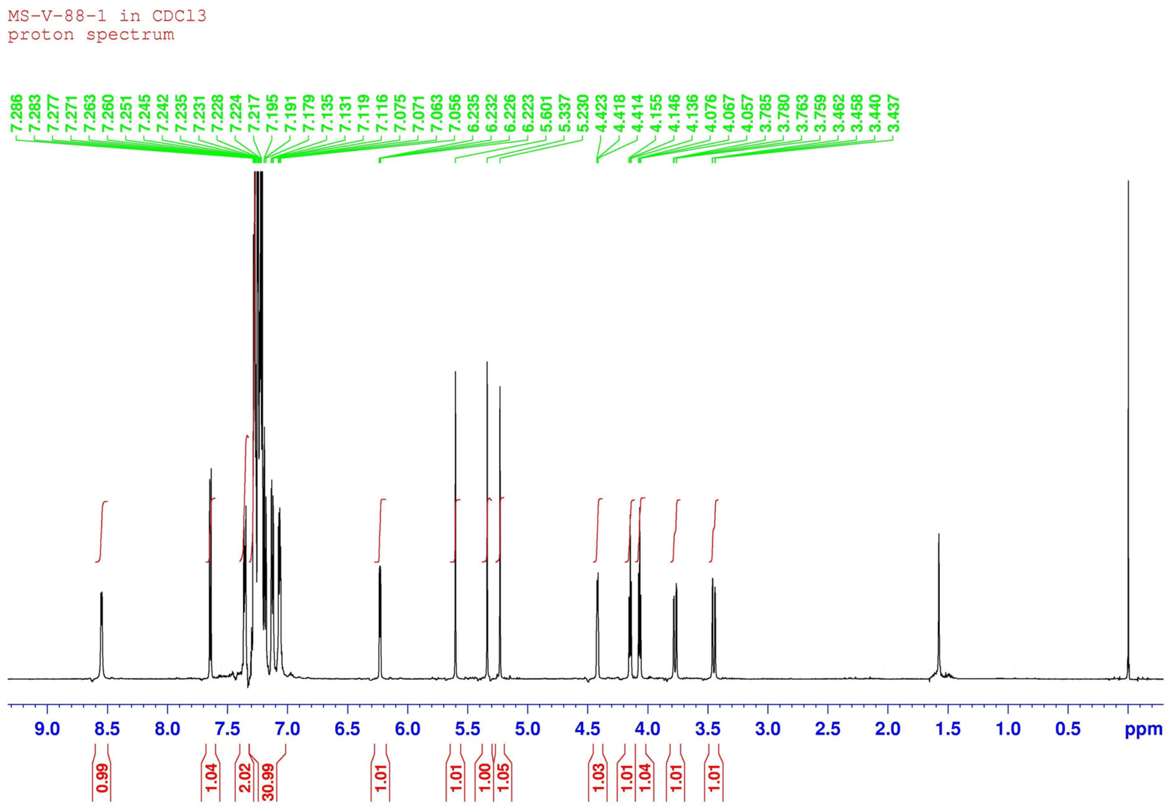

^{13}C-NMR (150 MHz, $CDCl_3$)

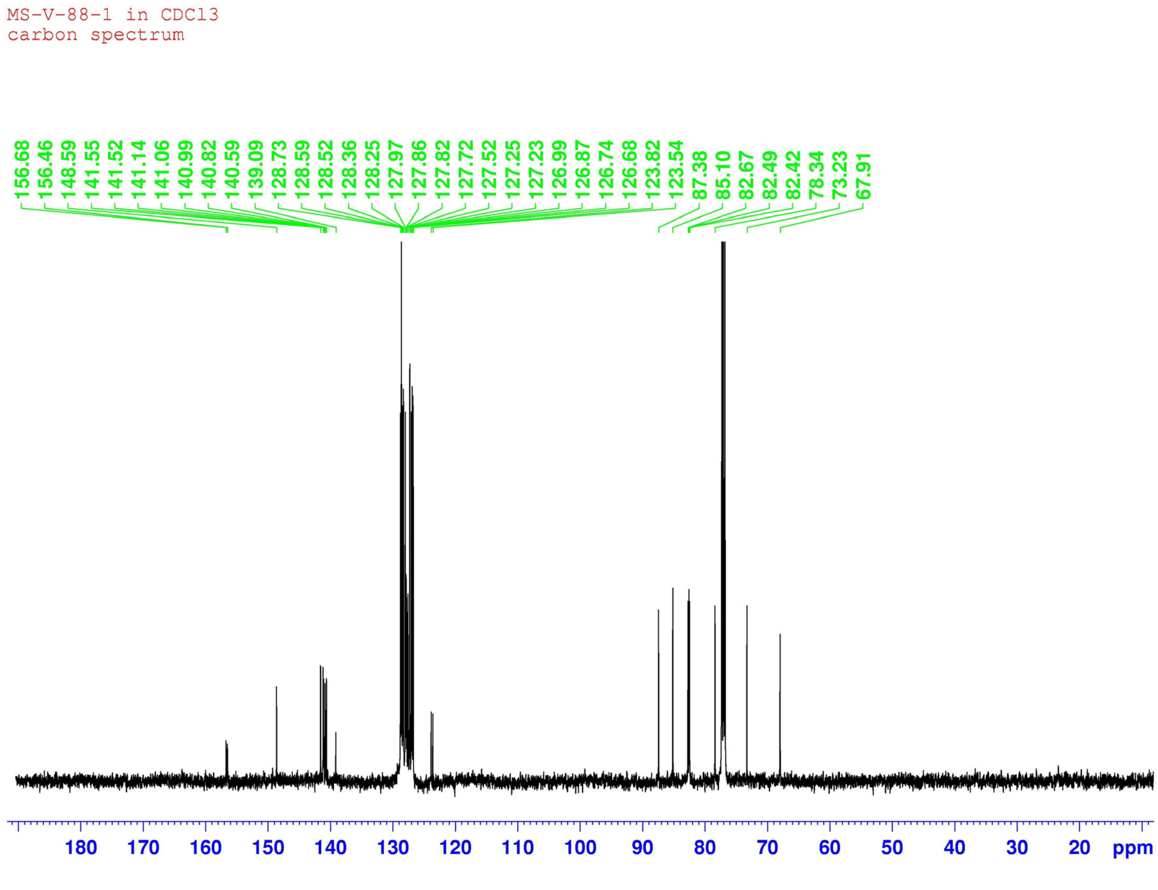

Figure S11. ^{1}H and ^{13}C-NMR Spectra of Ethoxydiphenylmethane (5).

^{1}H-NMR (300 MHz, $CDCl_3$)

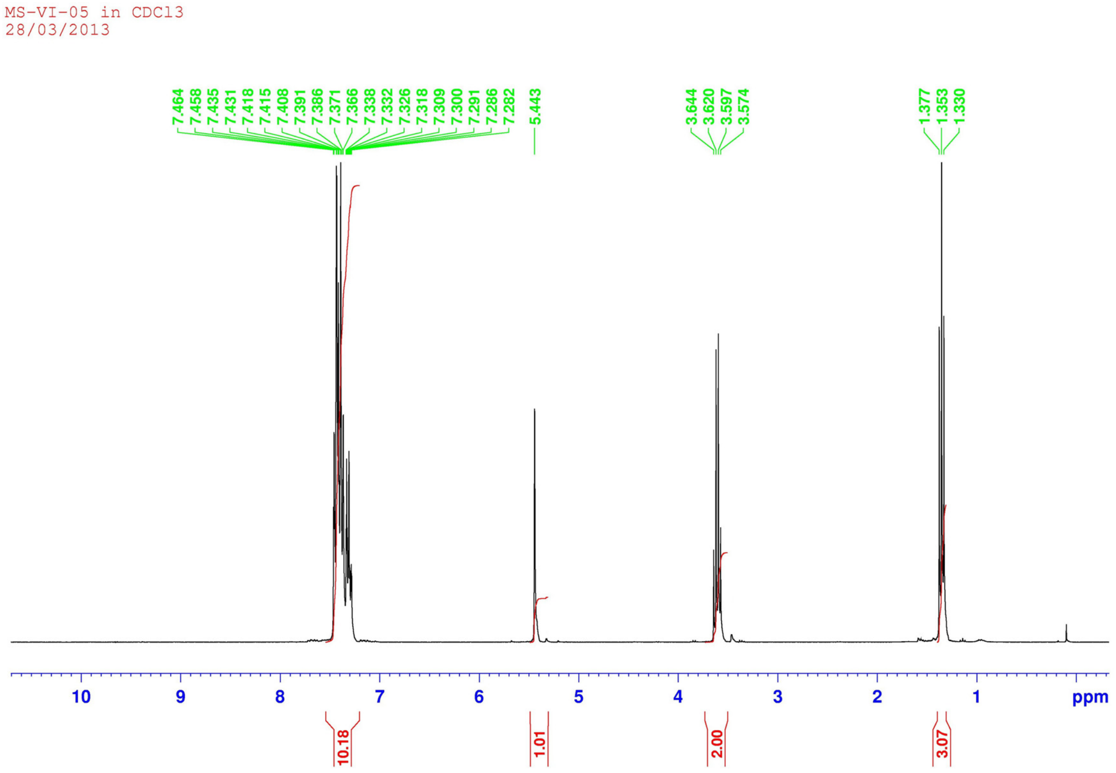

^{13}C-NMR (75 MHz, $CDCl_3$)

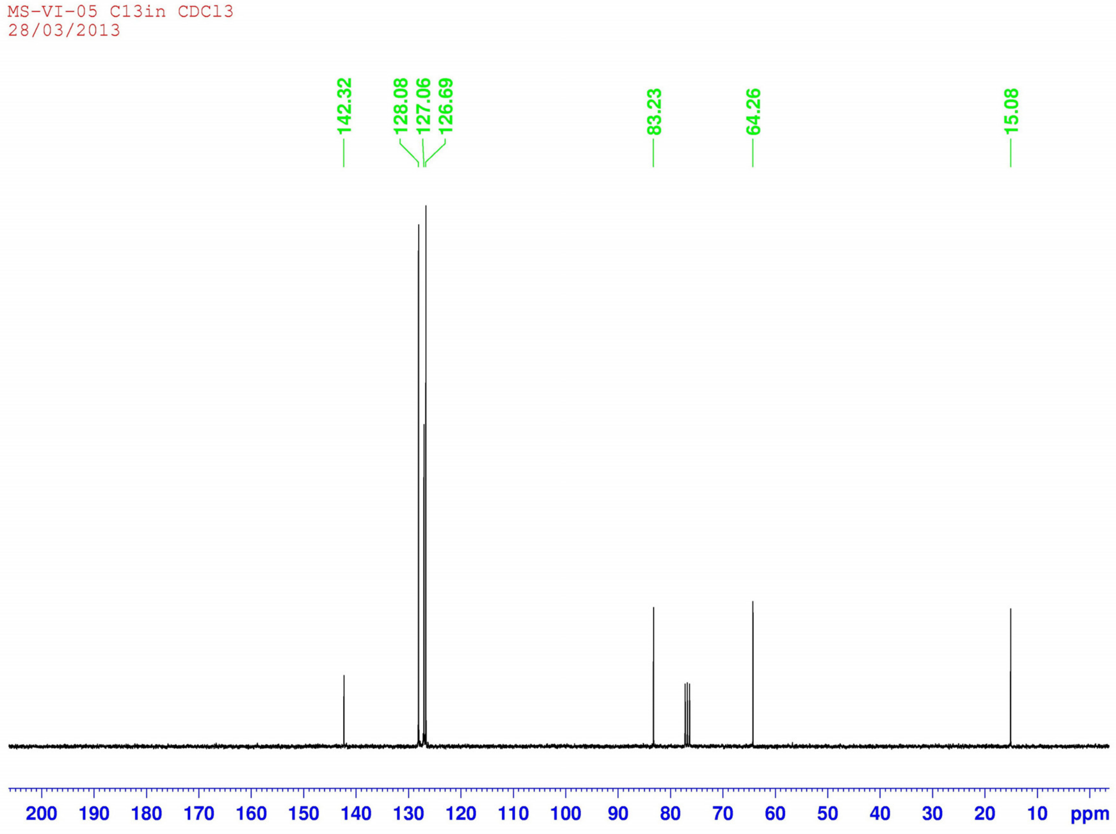

Figure S12. ^{1}H and ^{13}C-NMR Spectra of Diphenyl methyl ether (**4**).

^{1}H-NMR (300 MHz, $CDCl_3$)

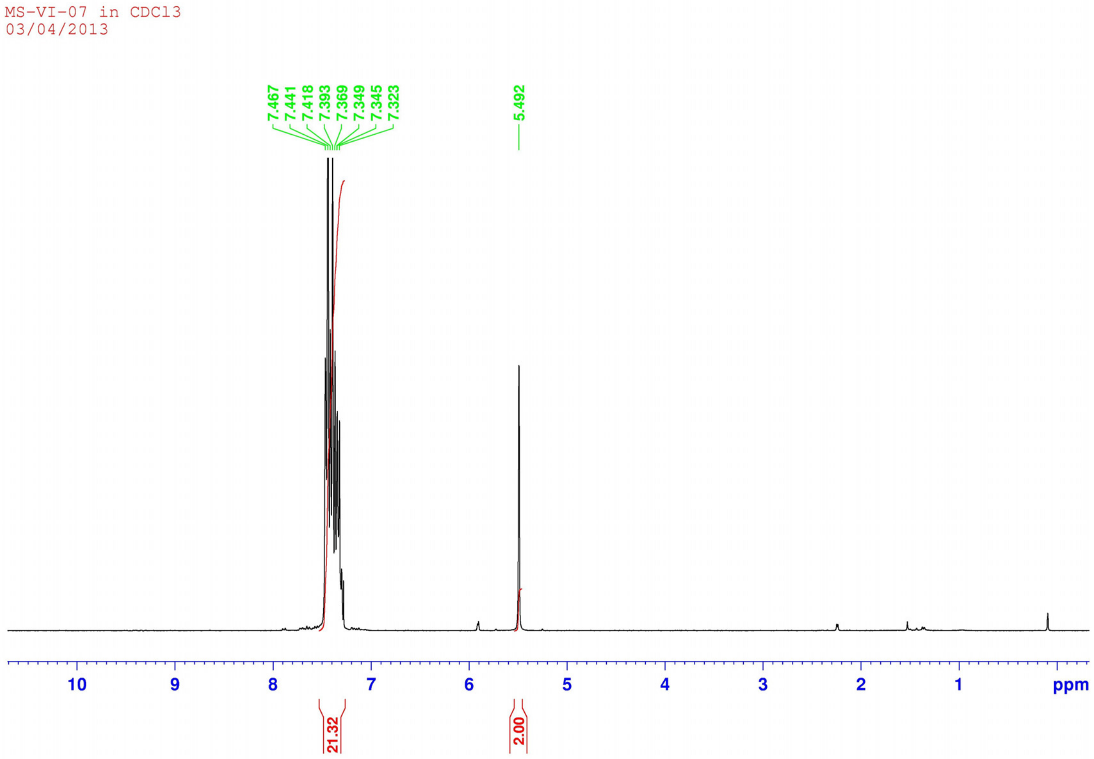

^{13}C-NMR (75 MHz, $CDCl_3$)

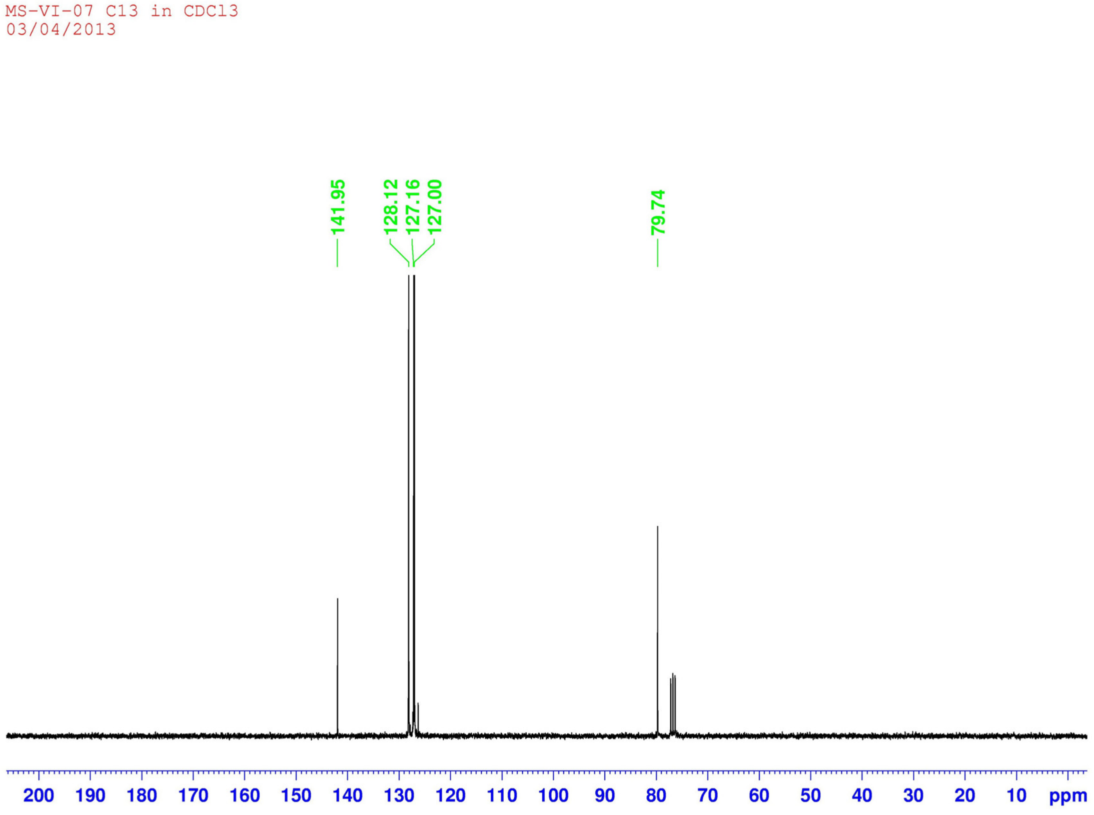

Supplement: Supplementary file 1 [file molecules-18-08524-s001.pdf]
